# Up, down, and all around? Deciphering the boundary conditions for training-induced transfer effects within a set of hierarchically nested tasks

**DOI:** 10.1177/17470218251334370

**Published:** 2025-04-02

**Authors:** Joseph Rennie, Duncan E Astle

**Affiliations:** 1 Department of Psychology, University of Exeter, Exeter, UK; 2 MRC Cognition and Brain Sciences Unit, University of Cambridge, Cambridge, UK; 3 Department of Psychiatry, University of Cambridge, Cambridge, UK

**Keywords:** Cognitive training, visual short-term memory, visual working memory, training transfer

## Abstract

Convergent evidence suggests that the transfer effects engendered by training studies are tied to specific task features. The present study examined transfer in a set of three hierarchically nested change-detection tasks (CDTs) using a tightly controlled adaptive training paradigm. These CDT paradigms all required participants to remember arrays of stimuli, and then report the change (in colour, orientation or both) of a probed item. The three tasks were identical except for the response judgement requirements: colour, orientation or dual (both colour and orientation). We also included a retro-cue – a spatial cue within the retention period – allowing us to test whether training impacts the allocation of attention during maintenance. Each training group made significantly greater on-task gains relative to the active control group (digit-span training). Between-task transfer patterns were present but limited and largely feature-specific. Training gains on the orientation task did not transfer to the colour variant and vice versa; in fact, there was some evidence of negative transfer. However, those trained on the colour variant did show benefits to both variants within the dual-task context. The dual-CDT trainees also showed transfer to the simple orientation and colour variants. Finally, we found no compelling evidence to distinguish whether training gains and transfer effects are due to improved capacity or the improved precision with which representations are held. In short, participants learn to better represent and report specific features, but it is not clear if this is driven by changes in capacity or precision.

## Introduction

Cognitive training refers to a variety of techniques and exercises designed to enhance cognitive abilities such as memory, attention, problem-solving and reasoning. The basic premise is that extended practice – sometimes called training – on one or more cognitive tasks improves performance on other, unpractised, tasks or activities that rely upon shared processes (Green & Bavelier, 2008; Simons et al., 2016; Taatgen, 2013). Researchers typically use a set of ‘assessment’ tasks believed to tap certain aspects of cognition, deployed before and after practice on a different set of ‘training’ tasks. When practice improves performance on another unpractised task, this is taken as evidence of generalisation, implying that something learnt in one context carries over or ‘transfers’, to another. The gold-standard design is to compare these effects against an appropriate active control condition. There are many reasons why the use of an active control group is important. An obvious reason to include an active control is that without one it is impossible to know if any improvements are specifically due to the *training itself* and not just practice on the assessments themselves (Simons et al., 2016). However, there are multiple other good reasons to include an *active* control group, such as controlling for general arousal, motivational or reward processing differences that might exist between training and inactive control groups (Green et al., 2014).

Due to the far-reaching implications for theory, education and wellbeing, multiple studies over recent decades have tested training-induced transfer effects (Green & Bavelier, 2008; Sala & Gobet, 2019; Simons et al., 2016). As has been reviewed extensively in the preceding literature, there is plenty of evidence for ‘near’ transfer, but ‘far’ transfer remains controversial (Au et al., 2015; Gathercole et al., 2019; Green & Bavelier, 2008; Holmes et al., 2009; Jaeggi et al., 2008; Klingberg, 2010; Melby-Lervåg et al., 2016; Sala & Gobet, 2019; Simons et al., 2016; Smid et al., 2020; Soveri et al., 2017). That is, training improvements often carry over to similar tasks within a domain but rarely translate to improvements in more distant tasks. Moreover, even within domain improvements appear bound to specific task features and may even fail to transfer between two tasks that differ by only a single feature (Gathercole et al., 2019; Rennie et al., 2021).

There remains no generally accepted method for defining how tasks relate to one another and no taxonomy by which to determine the extent to which two given tasks overlap, and thus to know how ‘near’ or ‘far’ they are from one another. Consequently, it is difficult to pin down precisely which processes are targeted by, or manifest because of, training and any boundary conditions for their transfer (Gathercole et al., 2019; Simons et al., 2016). Recent studies highlight the prevalence of feature-specific improvements that occur following typical cognitive training paradigms (Gathercole et al., 2019; Holmes et al., 2019; Norris et al., 2019; Rennie et al., 2021). As such, there is a call for more systematic and tightly controlled manipulations of task features to better establish potential boundary conditions of transfer and advance cognitive theory (Gathercole et al., 2019; Holmes et al., 2019; Katz et al., 2018; Norris et al., 2019; Redick, 2019; Sala & Gobet, 2019; Taatgen, 2013; von Bastian & Oberauer, 2014). That is, using tasks in both the assessment and training phases that are nested and vary systematically with respect to specific external task features, may allow researchers to infer more precisely the underlying mechanisms (Holmes et al., 2019; Norris et al., 2019; Rennie et al., 2021). The change-detection task (CDT) is a popular measure of visual working memory (VWM) that lends itself nicely to a nested approach for both practical and theoretical reasons. There exists a rich theoretical backdrop pertaining to both the CDT tasks themselves and the training context. In this study, we use nested CDT variants to investigate which skills are acquired during training and whether these are feature-specific or generalise more broadly.

### VWM and the CDT

VWM can be conceptualised as the limited capacity ability to maintain and manipulate visual information over short periods of time, in service of ongoing task demands (Baddeley & Hitch, 1974; Barak & Tsodyks, 2014; Christophel et al., 2017; Cowan et al., 2005; Luck & Vogel, 2013). VWM ability is of great interest because it strongly correlates with measures of cognitive control, intelligence and educational attainment, as well as being essential for successfully performing many everyday tasks (Alloway & Alloway, 2010; Cowan et al., 2005, 2006; Diamond, 2012; Fukuda et al,. 2010; Johnson et al., 2013; Luck & Vogel, 2013).

The CDT paradigm is a popular measure used to tap VWM (Buschkuehl et al., 2017; Luria & Vogel 2011). There are several variations of the CDT paradigm, each providing useful insights in its own right. However, they all follow the same general form: a brief presentation of a memory array containing a set of visual stimuli, followed by a retention interval, and then a test array containing one or more probe stimuli. Participants are required to judge whether (or how) some aspect of a probe stimulus has changed relative to its counterpart (e.g. same location) in the memory array. Conceptually, these three phases correspond roughly to: encoding, maintenance and retrieval (Buschkuehl et al., 2017; Fougnie & Alvarez, 2011; Luck & Vogel 1997).

A seminal study conducted by Luck and Vogel (1997) investigated VWM using a series of CDT paradigms. They found that participants had a high accuracy for detecting changes in a single feature dimension (e.g. colour) for up to four objects, with a sharp decline thereafter. Crucially, they also found that participants had an almost identical accuracy for detecting changes across multiple feature dimensions (e.g. colour and orientation). They took this as evidence to suggest that VWM stores discrete object-level representations rather than individual features and has a capacity of 3 to 4 items. Others too have proposed that VWM consists of a small number of slots, each of which stores a single integrated visual object, or chunk, with fixed precision (slot model; Cowan, 2001; Luria & Vogel, 2011; Rouder et al., 2008; Vogel et al., 2001; Zhang & Luck, 2008). However, subsequent lines of research have questioned both the proposed capacity estimates and the nature of VWM representations (Alvarez & Cavanagh, 2004; Bays et al., 2009; Schneegans & Bays, 2016; Souza & Oberauer, 2016).

Continuous response paradigms allow researchers to examine the precision of recall. As the number of items in the memory array (set size) increases, response precision decreases exponentially, indicating that the resolution with which representations are stored trades off against the number of items being stored (Alvarez & Cavanagh, 2004; Awh et al., 2007; Bays & Husain, 2008; Bays et al., 2009; Wilken & Ma, 2004). These findings appear consistent with a model of VWM in which the precision of a stored representation depends upon the proportion of a common VWM resource allocated to it (resource model; Bays & Husain, 2008; Bays et al., 2009). Participants do not know which item from the memory array will be probed, and will therefore share resources amongst the items, hence why performance declines as the number of items in the memory array increases.

Importantly, CDT paradigms require memory not only for the to-be-reported dimension (e.g. colour or orientation) but also for the cue dimension (e.g. location). This can sometimes lead to what are called ‘swap errors’, errors thought to occur when features of non-target items interfere with the recall of the target item due to a failure in correctly integrating, or ‘binding’, information across the report and cue dimensions (Schneegans & Bays, 2016, 2017). Others have found that when participants are required to recall two cue features, such as colour and orientation, from objects in a memory array within a continuous response paradigm, the errors for each of the features are weakly correlated with one another and thus appear to be relatively independent (Bays et al., 2011; Fougnie & Alvarez, 2011). Relatedly, others have found a monotonic decrease in CDT performance as the complexity of objects in the memory array increases (Alvarez & Cavanagh, 2004; Olson & Jiang, 2002; Wheeler & Treisman, 2002; Xu, 2002). Together, the above findings imply that VWM resources are allocated, at least somewhat independently, to features within a visual scene rather than objects.

Marshall and Bays (2013) argue that by attending to one feature, all others are automatically encoded, but that maintenance of specific features can be modulated by attention. This sentiment is echoed by the well-established retro-cue effect. A retro-cue is an attentional cue presented during the maintenance phase of a trial, providing an indication as to which item will be probed at retrieval, and thereby orienting attention to the internal representation of that item (Griffin & Nobre, 2003; Souza & Oberauer, 2016). Retro-cues can substantially improve VWM performance in terms of both accuracy and reaction time (Astle, Summerfield et al., 2012; Griffin & Nobre, 2003; Heuer & Schubo, 2016; Sligte et al., 2008, 2010; Souza & Oberauer, 2016). This suggests that VWM capacity is greater during maintenance than no-cue trials alone would suggest, but that it is negatively impacted by retrieval and/or response processes (fragile memory). It also provides a powerful demonstration of the important role that top-down attention plays in modulating the content of VWM (Griffin & Nobre, 2003; Heuer & Schubo, 2016; Oberauer & Hein, 2012; Souza & Oberauer, 2016). By allowing VWM resources to be flexibly allocated in time and space, visually salient items may be remembered with enhanced precision and protected from interference, whilst less important items may be weakened, thereby freeing up resources for use elsewhere (Astle et al., 2012; Heuer & Schubo, 2016; Marshall & Bays, 2013; Souza & Oberauer, 2016).

Whilst the capacity and nature of VWM are contentious, there is evidence to suggest that VWM resources may be flexibly allocated via attention. As such, training people on CDT paradigms may lead to changes in their attention to certain features, thereby making them more or less salient. By including several CDT training variants, we are able to ask whether any changes in attention occur, and if so, are they feature specific or do they generalise more broadly? Moreover, including retro-cues on half of the assessment task trials enables one to test whether these training effects impact upon the spatial allocation of attention during the maintenance phase, or alternatively, whether they impact performance independently of this.

### Change-detection training

Despite its popularity, only a handful of studies to date have looked at the effects of training on the CDT paradigm. This is perhaps due to a few early studies reporting only small effects for on-task improvements following practice (Olson & Jiang, 2004; Olson et al., 2005). However, these studies were primarily investigating the effect of long-term memory traces within sessions, rather than transfer effects, and so were necessarily limited in scope. More recently, Xu et al (2018) found small-to-moderate improvements across 30 sessions of training on a CDT paradigm and found performance to be relatively stable over time with respect to both within and between subject performances. However, this training was non-adaptive. Adaptive training (that which increments in difficulty as participants improve at a task) is thought to enhance training gains above and beyond non-adaptive training (Buschkuehl et al., 2017; Jaeggi et al., 2014). Using an adaptive training regime Buschkuehl et al. (2017) found substantial on-task improvements following 4 hr of colour-CDT training (20%–25% increase in Cowan’s K, a measure of the number of correct items, taking into account set size and false alarm rate). They also found very little indication of transfer across several other measures, including a colour resolution task and a similarly structured CDT paradigm that used more complex stimuli. They suggest that the training likely had an impact on early stimulus representations, specific to the task, but not on the ability to retain details of the stimuli or any higher-level cognitive processes. Moreover, the study did not contain a control group, and thus it cannot control for any test-retest effects.

Only a few studies have used a control group, and they have found mixed results (Gaspar et al., 2013; Moriya, 2019; Norris et al., 2019). Gaspar et al (2013) trained participants adaptively on an object-CDT paradigm by progressively reducing the display time of the memory array, encouraging faster encoding. This training substantially improved on-task performance, but these improvements did not transfer to their ‘near-transfer’ measure of a similar object-CDT paradigm, nor to their ‘far-transfer’ measure of a flicker-CDT paradigm. The authors concluded that the training improvements were due to an increased familiarity with the training stimuli rather than an improvement in any change-detection ability more broadly. However, as the authors note, their training targeted processes associated with faster encoding of the stimuli so we do not know whether training aimed at other processes associated with capacity may have produced a different outcome. Moreover, despite labelling one of the assessment tasks as a ‘near-transfer’ measure, it still differed from the training task with respect to timings, masking, stimulus categories and set size manipulations, thus making it difficult to establish precisely what constrained transfer.

Alternatively, Norris et al. (2019) trained participants adaptively on a colour-CDT paradigm by progressively increasing the number of items in the memory array (set size), encouraging enhancements in capacity. This training substantially improved on-task performance and these improvements transferred to an untrained orientation-CDT paradigm that was identical to the training task, except the memory items were oriented bars instead of coloured squares. This suggests that at least some of the cognitive processes acquired during colour-CDT training are generalisable, such that they can be utilised across stimulus types. In contrast, Adam and Vogel (2018) found feature-specific transfer after training participants on a colour-whole-report task paradigm (‘whole-report’ means that participants report their memory of the actual stimuli, not just whether it has changed). Specifically, they found that colour-whole-report training transferred to a colour-CDT paradigm (same feature modality, different response requirement) but not to an orientation-whole-report task (different feature modality, same response requirement). However, the training used here was not adaptive. Moreover, the response requirements of whole-report tasks are a lot more involved compared with those in the CDT, and so any transfer benefits occurring at encoding or maintenance may have been ‘washed out’ due to the interference caused by the response requirements.

Another recent study by Moriya (2019) trained participants on two highly similar orientation-CDT paradigms and tested participants on equivalent assessment versions of these tasks before and after training. One task was considered a ‘quantity task’ and the other as a ‘quality task’. The quantity task contained set sizes of 4, 6 and 8, with probe offsets of 45°, whilst the quality task contained set sizes of 2, 4 and 6, with probe offsets of 15°. As in Norris et al. (2020), participants trained adaptively by progressively increasing the set size of the memory array. Moriya found that training on the quantity version of the task transferred to the quality version but not the other way around. Moriya interpreted this finding as support for the idea that training enhances the allocation of limited VWM resources for both the quantity and quality of the memory items and that the two share overlapping processes. However, if this were so, it is unclear as to why we would see a transfer one way but not the other (although it was trending in that direction and may simply be a power issue). Moreover, the nature of the quality training was adaptive for set size and not offset, thus it could be argued that it was also training quantity but just at smaller offsets. An alternative interpretation of these results is that the quality task is a watered-down version of the quantity task, with higher variance within the set size due to the increased difficulty provided by the smaller offset. Nonetheless, the positive transfer found in this study is further evidence that skills acquired during CDT training may generalise to similar contexts. However, given that offset difficulty was not varied systematically at assessment, we do not know whether the transfer effect found here represents an improvement in perceptual accuracy, an increase in the ability to store more objects in memory or both (Bays & Husain, 2008; Bays et al., 2009).

The CDT appears to be an underexplored paradigm in the context of training, and thus the boundary conditions for transfer within this otherwise popular working memory paradigm remain unclear. Given the substantial on-task training gains following adaptive set size training (Buschkuehl et al., 2017; Moriya, 2019; Norris et al., 2020), the potential of transfer to similarly structured variants (Moriya, 2019; Norris et al., 2020), and the unresolved mechanisms of improvement (e.g. quantity vs. quality), the current training study appears promising and well situated to further inform these areas.

### Overview

There is increasingly convergent evidence to suggest that the transfer engendered by typical training studies is tied to specific task features (Gathercole et al., 2019; Melby-Lervåg et al., 2016; Norris et al., 2019; Sala & Gobet, 2019; Simons et al., 2016). The CDT paradigm lends itself well to subtle feature manipulations, which have proved fruitful in furthering our understanding of VWM (Alvarez & Cavanagh, 2004; Bays et al., 2009; Luck & Vogel, 1997, 2013; Luria & Vogel 2011; Schneegans & Bays, 2016; Souza & Oberauer, 2016). Whilst the nature of VWM and its representations remain contentious, there is evidence to suggest that VWM resources can be flexibly allocated via attention to aid task performance (Griffin & Nobre, 2003; Heuer & Schubo, 2016; Luck & Vogel, 2013; Marshall & Bays, 2013; Schneegans & Bays, 2017; Souza & Oberauer, 2016). However, due to the limited number of randomised controlled training studies using the CDT paradigm, it remains unclear just how malleable the VWM resources used to perform the CDT are, or the extent to which changes that occur as a result of experience are tied to specific features or generalise more broadly. Given the potential for on-task training gains (Buschkuehl et al., 2017; Xu et al., 2018), transfer between similarly structured variants (Moriya, 2019; Norris et al., 2020), as well as a rich theoretical backdrop, the CDT paradigm provides a good training context for exploring the relationship between task overlap and training transfer.

### The present study

**Figure 1.**
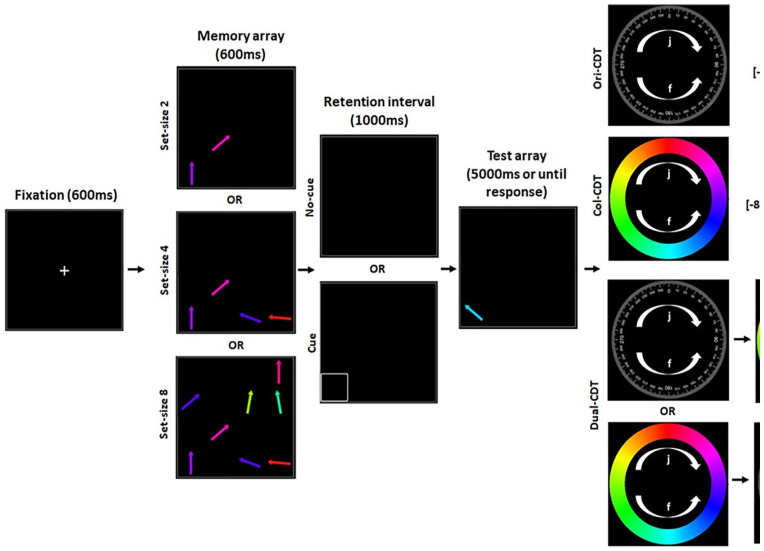
CDT trial flowchart. Trial structure for the three CDT variants. Participants were presented with a memory array containing a number of arrows (2, 4 or 8) of various colours and orientations, followed by a retention interval, and then a test array containing a single probe stimulus. The probe stimulus was always offset relative to its counterpart (same locations) in the memory array, with respect to both its colour and orientation, by either a small, medium or large degree. In each of the three tasks, participants were required to judge the circular direction (clockwise or anticlockwise) of change for either the colour, orientation or both the colour and orientation of the arrows, respectively. Participants were instructed to press the ‘J’ key when making a clockwise response or the ‘F’ key when making an anticlockwise response. They were instructed to be both accurate and fast. On the CDT assessment tasks, half of the trials included a retro-cue in the second half of the retention interval, cueing participants toward the location of the stimulus to be tested. Participants were provided feedback for 300 ms after each probe response by the reference wheel turning green and displaying ‘correct’ for a correct response or turning red and displaying ‘incorrect’ for an incorrect response. The training versions of these tasks were identical except they did not contain any retro-cues and the set size was adjusted adaptively in response to the participant’s performance. *Note*. CDT = change-detection task.

The present study investigated which skills are acquired during adaptive training on a set of three hierarchically nested CDTs and the potential boundary conditions for the transfer of these skills. To do so, we conducted a large online training study, powered for small-to-medium effect sizes. We used three CDTs that were structurally almost identical but varied subtly from one another with respect to their specific judgement requirements. We also used a digit-span task as an active control condition. There were assessment versions of the four tasks and each also had a training version counterpart. Participants completed 12 sessions of training, which were adaptive with respect to the set size of the memory array (span length in the digit-span training). In all three CDT tasks, participants were presented with a memory array containing a number of arrows of various colours and orientations, followed by a retention interval, and then a test array containing a single probe stimulus. Importantly, the probe stimulus was always offset relative to its counterpart (same locations) in the memory array, with respect to both its colour and orientation, by either a small, medium, or large degree. In each of the three CDT tasks, participants were required to judge the circular direction (clockwise or anticlockwise) of change for either the colour, orientation or both the colour and orientation of the arrows, respectively (see Figure 1). On the CDT assessment tasks, half of the trials included a retro-cue in the second half of the retention interval, cueing participants toward the location of the stimulus to be tested.

This design was motivated by, and allowed us to ask, the following questions, along with the corresponding predictions (pre-registered: https://osf.io/nb5m7):

1. Does training lead to the acquisition of skills that enhance the number of items stored in memory, the precision of those items, or both?

Given the evidence for the modulation of both the quantity (Buschkuehl et al., 2017; Griffin & Nobre, 2003; Heuer & Schubo, 2016; Moriya, 2019; Norris et al., 2020) and quality (Bays et al., 2009; Heuer & Schubo, 2016; Marshall & Bays, 2013; Moriya, 2019) of VWM representations via spatial attention and experience, we predicted that the training would enhance on-task performance in terms of both the number of items held in memory and the precision of the representations of those items. However, we acknowledged that these two factors interact with one another so this may be hard to tease apart (Alvarez & Cavanagh, 2004; Bays & Husain, 2008; Bays et al., 2009).

2. Does training affect the allocation of spatial attention during VWM maintenance?

We speculatively predicted that training gains will have a greater impact on processes at the encoding/early maintenance phase of the trial due to the relatively long presentation times used here, leaving more room for strategically improving encoding efficiency, especially at higher set sizes (Gaspar et al., 2013; Vogel et al., 2006) and evidence suggesting an increased value of attentional modulation at encoding/early maintenance phases of a trial versus later on (Astle et al., 2012; Griffin & Nobre, 2003). If this is so, then we should not expect training to interact with the presence of the retro-cue during the post-encoding maintenance phase. Alternatively, if extended CDT practice essentially trains participants to allocate spatial attention strategically during the maintenance phase, then training should interact with the retro-cue/no-cue manipulation.

3. Are the skills acquired during training in the single judgement tasks bound to their specific judgement types of colour and orientation, or do they transfer to one another?

If maintaining representations with increased precision requires more focused attention (Alvarez & Cavanagh, 2004; Bays & Husain, 2008; Bays et al., 2009; Oberauer & Hein, 2012) and memory for within-object features can fail independently (Bays et al., 2011; Fougnie & Alvarez, 2011), then training in the single feature conditions may encourage a greater saliency for specific features, and thus we predicted that any training gains *for quality* of the representations will not transfer across judgement types. On the other hand, given that maintaining a greater number of items appears to require a broader focus of attention (Alvarez & Cavanagh, 2004; Bays & Husain, 2008; Bays et al., 2009; Oberauer & Hein, 2012), wherein features of the objects appear to be bound to a certain extent (Luck & Vogel, 2013; Luria & Vogel, 2011), and the prior evidence for the transfer of quantity gains across judgement types (Norris et al., 2020), we predicted that training gains *in quantity* will generalise across judgement types. In short, we predicted that there would be some lateral transfer from one judgement type to another.

4. Do the skills acquired during training in the single task conditions transfer ‘up’ the task hierarchy to a dual judgement (Orientation and Colour) task and vice versa?

We did not make any specific predictions at pre-registration about the vertical direction of transfer specifically. However, it would seem reasonable, based on the findings from Rennie et al. (2021), to suggest that a varied exposure of features at a higher level may encourage broader transfer, and thus that dual judgement training would transfer to both of the lower-level tasks, whilst single feature conditions may be constrained more specifically and not transfer ‘up’.

## Materials and methods

### Ethical approval

This study received ethical approval from the Cambridge Psychology Ethics Committee, University of Cambridge, application number: PRE.2019.046. All participants provided informed consent by checking a box to confirm they had fully understood the implications of participation and their right to withdraw.

### Participants

The final sample herein (see section ‘Data exclusion’), consisted of 168 English-speaking adults with normal/corrected vision aged between 18 and 35 years of age. Participants were recruited via ‘Prolific’, a platform for recruiting and paying people to participate in online experiments. Participants were paid at a rate of £6 per hr and received an £8 bonus upon the satisfactory (i.e. not suspect of low effort or cheating) completion of all sessions. Participants were randomly assigned to one of the four training conditions: Orientation-CDT (*N* = 42), Colour-CDT (*N* = 41), Dual-CDT (*N* = 41) and Digit-Span (control, *N* = 44). All participants completed two assessment sessions, one before (pre) and one after (post), completing 12 sessions of adaptive training.

### Assessment tasks and procedure

In each assessment phase (pre- and post-training) participants completed all four assessment tasks: (a) Colour Change-Detection Task (Col-CDT); (b) Orientation Change-Detection Task (Ori-CDT); (c) Dual Change-Detection Task (Dual-CDT), split into two response types of Orientation (Dual-Ori-CDT) and Colour (Dual-Col-CDT) and (d) Digit-Span (DS) Task. Each assessment phase took approximately 90 min to complete. In both sessions, participants first completed the single-response Col-CDT and Ori-CDT in a randomised order, followed by the dual-response (Colour & Orientation) Dual Change-Detection Task, and finally the DS task. All tasks were coded using JavaScript (jsPsych; de Leeuw, 2015), HTML and CSS in house. Provided below are detailed written accounts of each of the task materials and procedures. Also, see Figure 1 for a graphical depiction of the CDT task trials.

### Further detail on CDTs

All three CDTs required participants to make two alternative forced choices (2AFC) about the direction in which a probe stimulus had changed relative to its counterpart in the memory array presented prior to a retention interval. In each, participants were required to make these judgements according to changes in orientation, colour or both. Participants were instructed to press the ‘J’ key when making a clockwise response or the ‘F’ key when making an anticlockwise response. They were instructed to be both accurate and fast. Participants received explicit step-by-step instructions along with examples and a small number of practice trials for each of the tasks. Attention checks were in place following each set of task instructions to help ensure that participants understood what was required; these were in the form of multiple-choice arrays containing each of the judgement requirements, participants had to choose the appropriate judgement to continue with the task, a failure to do so resulted in a repetition of the task instructions. Participants were provided with eight simple practice trials; a failure to score above a certain accuracy threshold (70% and 60% for the single-response and dual-response tasks respectively) resulted in a single repetition of the practice trials. Again, this was to help ensure participants understood the requirements and response mappings before starting the task. We considered this especially important in this study due to its online nature and the high similarity between tasks. Participants completed two blocks on each of the three CDT tasks, with a short break in between each block. Participants received immediate feedback after each trial and statistics about their overall performance (mean accuracy and reaction time (RT)) at the end of each task. All the CDT assessment tasks followed the same general form in terms of their procedural structure, timings and the stimuli presented, the order and content of the phases in any given trial were as follows: fixation (600 ms), memory array (600 ms), retention interval (1,000 ms), test array (maximum 5,000 ms per response), feedback (300 ms), inter-trial interval (500 ms). The details of the stimuli presented in each of these phases are outlined below (also see Figure 1).

### Fixation

A central fixation cross with a font size of 100 px is presented for 600 ms within a 600 px × 600 px grid square.

### Memory array

An evenly spaced 4 × 4 grid square (grid-height: 600 px, grid-width: 600 px; cell-height: 150 px, cell-width: 150 px) containing either 2, 4 or 8 coloured arrows was presented for 600 ms. Each arrow was 120 px in length with a linewidth of 13 px and an arrowhead-width of 21 px. Each arrow was positioned centrally within a random cell, orientated randomly about a circular space divided evenly into increments of 5 angular degrees (360/5 = 72 potential starting orientations), and coloured randomly about a Hue Saturation Lightness geometric colour space, with hue mapping to a circular space and divided evenly into increments of 5 angular degrees (360/5 = 72 potential starting hues), whilst saturation and lightness were held constant at 100% and 50% respectively.

### Retention interval

Trials were divided evenly into retro-cue and no-cue trials. On no-cue trials, a blank 600 px × 600 px grid square was presented for 1,000 ms. On retro-cue trials, a blank 600 px × 600 px grid square was presented for 500 ms, followed by a 600 px × 600 px grid square presented for 500 ms in which one of the grid cells was outlined, cueing the participant to the relevant target location for the upcoming probe stimulus.

### Test array

On each trial, one of the cells from the memory array was selected at random as a target location to be probed following the retention interval. The arrow from the memory array at the selected location was transformed according to both its orientation and colour: the orientation of the arrow was offset by either −40°, −15°, −6°, 6°, 15° or 40°; likewise, the colour of the arrow was offset by either −80°, −30°, −15°, 15°, 30° or 80°. These increments were determined by extensive piloting of the tasks to try and achieve equivalent difficulty. The transformed arrow was presented at the same location within the same 4 × 4 display grid until the participant gave a response or 5,000 ms had elapsed. A failure to respond within 5,000 ms was counted as incorrect. For orientation judgements, participants were provided with a reference orientation wheel to the right of the display grid, which contained directional arrows reminding the participant of the appropriate response options. Likewise, for colour judgements, participants were provided with a reference colour wheel to the right of the display grid, which contained directional arrows reminding the participant of the appropriate response options, and crucially, the colour mappings onto the circular space. Participants were required to make a 2AFC judgement about the direction in which the probe arrow had changed according to either its colour or orientation, relative to its counterpart arrow (same location) in the memory array. They were instructed to press the ‘J’ key for a clockwise response or the ‘F’ key for a counterclockwise response and to be both accurate and fast. In the dual-response task, a probe display grid was shown first requiring one judgement type and then followed by the other (i.e. colour then orientation or orientation then colour).

### Feedback

Participants were provided feedback for 300 ms after each probe response by the reference wheel turning green and displaying ‘correct’ for a correct response or turning red and displaying ‘incorrect’ for an incorrect response.

### Inter-trial interval

After each trial, a blank 600 px × 600 px grid square was presented for 500 ms.

### Task variants

Whilst all the CDT tasks followed the same general form, what differentiated them were the required judgements about the probe arrow relative to its counterpart in the memory array grid (arrow in the same cell location). The different tasks and their judgement requirements are outlined below (see Figure 1 for a graphical depiction).

### Orientation-CDT

This task required participants to respond specifically to changes in the orientation of the probe arrow relative to its counterpart in the memory array.

### Colour-CDT

This task required participants to respond specifically to changes in the colour of the probe arrow relative to its counterpart in the memory array.

### Dual-CDT

This task required participants to respond to changes in both the orientation and colour of the probe arrow relative to its counterpart in the memory array. To counterbalance the order of the response judgements (i.e. colour then orientation or orientation then colour) participants were presented with two variants of this task: in one they were required to first make a judgement about the changes in colour before making a second judgement about the changes in orientation; in the other, they were required to first make a judgement about the changes in orientation before making a second judgement about the changes in colour. From the participants’ perspective, these were presented as separate tasks, each with their own instructions and practice trials.

Although the stimuli parameters and trial orders were randomly generated for each CDT task independently, in the assessment tasks these were the same for all participants and at each assessment point (pre- and post-training). Trials were divided evenly according to set size, cue type and offset. For both the single-response tasks, there were 180 trials total, split evenly into 90 retro-cue trials and 90 no-cue trials. Each cue type contained 30 trials at each set size. Each set size contained 5 trials at each offset value. For the dual-response task, there were 216 trials total (counterbalanced for response order and combined), split evenly into 108 cue trials and 108 no-cue trials. Each cue type contained 36 trials at each set size. Each set size contained 6 trials at each offset value.

### DS task

This task required participants to memorise sequences of randomly sampled numbers. A number of digits ranging from 0 to 9 were sampled at random without replacement according to the current sequence length. For sequences >10, the initial set of 10 digits was appended with a further set of randomly sampled digits ranging from 0 to 9 accordingly. Participants were instructed to memorise the digit sequences as best they could and input them into a text-response box in the order they were presented. Participants received explicit instructions along with an example. They were also given three practice trials at a set size of three to help ensure they understood the task and how to respond appropriately. All participants began with a sequence length of three and were presented with up to six trials at each sequence length (maximum of 20). Participants progressed to the next sequence length if they scored over 50% accuracy at a given length, the task ended when they failed to achieve this.

### Training tasks and procedure

Upon completion of the first assessment session participants were randomly allocated to one of four training conditions: (a) Colour change-detection training (Col-CDT training); (b) Orientation change-detection training (Ori-CDT training); (c) Dual change-detection training (Dual-CDT training); or (d) DS training. Each received specific instructions about the training phase of the study and was linked to a personalised ‘homepage’. This homepage contained information about the number of sessions they had completed and how long they had to wait before starting the next, it also provided a portal to the next session when available. Participants were only allowed to access the next session after 3 hr had elapsed following the completion of the previous one. This homepage also contained a portal to the second assessment session, which participants were able to access 3 hr after they had completed all training sessions.

Each of the four training tasks had an almost identical assessment task counterpart (see section ‘Assessment tasks and procedure’ for details). Although highly similar, the training tasks differed from the assessment tasks in the following ways: (a) they did not include any practice trials; (b) CDT training tasks did not contain any cue trials; (c) their difficulty was adaptive – that is, adjusted on the fly to match the performance of the participant. The difficulty level achieved by the end of one training session carried over to the next. Participants received trial-by-trial feedback, level-up/down notifications, as well as feedback about the difficulty level achieved at the end of each session. The training stimuli parameters were unique to each participant, although, they followed the same parameter distributions as one another and their assessment task counterparts (i.e. same orientation/colour starting range, same orientation/colour offset range, same digit range). All participants received and completed 12 sessions of adaptive training in total. The unique details of each training task and condition are outlined below.

### Ori-CDT training

This task was structurally identical to the Ori-CDT assessment task, except the set size varied adaptively according to the participant’s performance level, and it did not contain any retro-cue trials. Each training session consisted between 200 and 212 trials (variable due to potential level-ups before the end of a block) and lasted approximately 15 to 17 min, with the opportunity for a short break halfway through. All participants started on the easiest difficulty level (set size of 1) in the first session, training difficulty (set size) was then adapted using a staircase procedure, if participants scored ⩾75% correct over 12 trials they were moved up a difficulty level (an increase in set size by 1) but if they scored ⩽58.3% correct over 12 trials they moved down a difficulty level (a decrease in set size by 1), otherwise they remained at the same difficulty level. These levels, and those for the other groups, were determined following extensive piloting of the training tasks.

### Col-CDT training

This task was structurally identical to the Col-CDT assessment task, except the set size varied adaptively according to the participant’s performance level and it did not contain any cue trials. Each training session consisted of between 200 and 212 (variable due to potential level-ups before the end of a block) trials and lasted approximately 18 to 20 min, with the opportunity for a short break halfway through. All participants started on the easiest difficulty level (set size of 1) in the first session, training difficulty (set size) was then adapted using a staircase procedure if participants scored ⩾75% correct over 12 trials they moved up a difficulty level (an increase in set size by 1) but if they scored ⩽58.3% correct over 12 trials they moved down a difficulty level (a decrease in set size by 1), otherwise they remained at the same difficulty level.

### Dual-CDT training

This task was structurally identical to the Dual-CDT assessment task, except the set size varied adaptively according to the participant’s performance level and it did not contain any retro-cue trials. Each training session consisted of between 200 and 212 trials (variable due to potential level-ups before the end of a block) and lasted approximately 20 to 25 min, with the opportunity for a short break halfway through. All participants started on the easiest difficulty level (set size of 1) in the first session, training difficulty (set size) was then adapted using a staircase procedure, if participants scored ⩾75% correct over 12 trials they moved up a difficulty level (an increase in set size by 1) but if they scored ⩽58.3% correct over 12 trials they moved down a difficulty level (a decrease in set size by 1), otherwise they remained at the same difficulty level. The difficulty level achieved by the end of one training session carried over to the next. The order of the response judgements (i.e. colour then orientation, or orientation then colour) alternated predictably between training sessions. In half the sessions, participants were required to first make a judgement about the changes in colour of the probe arrow before making a second judgement about the change in orientation of the probe arrow; in the other half, the order was reversed.

### DS training

Each training session lasted approximately 20 min (determined by a timer), with the opportunity for a short break halfway through. All participants started at the easiest difficulty level (sequence length of 1) for the first session, training difficulty (sequence length) was then adapted using a staircase procedure, if participants scored ⩾83.3% correct over 6 trials they moved up a difficulty level (an increase in sequence length by 1) but if they scored ⩽16.7% they moved down a difficulty level (a decrease in sequence length by 1), otherwise they remained at the same difficulty level. Again, these levels were determined following extensive piloting.

### Data exclusion

All incoming data were screened for quality based on summary statistics saved using JavaScript/JATOS. Participants with particularly low accuracy and reaction times across tasks at pre-training assessment (Accuracy <53% and RT <500 ms; based on pilot data) were assumed to not be engaging and excluded from the study. Furthermore, participants who did not complete all sessions were excluded from the analysis. Unfortunately, due to technical problems, there were issues retrieving the full data from the server. It is important to note that by including assessment versions of each training task, we were still able to test for on-task training effects. Of the 229 participants who started, 189 completed all sessions. Unfortunately, we were unable to retrieve the full assessment data (pre and post) for 19 (10%) of these participants so they were removed. Moreover, large chunks of training and demographic data were missing from all participants and as such we were not able to include training data or include full demographic information. After data collection, participants who scored below 2 standard deviations (calculated task-wise at pre-training) on two or more tasks at pre- or post-training were excluded from all subsequent analyses. Again, this was intended to remove participants who were not engaging with the tasks. This resulted in a further 2 out of the 170 participants to be excluded, leaving 168 participants for this set of analyses.

An a priori power analysis estimated that a sample size of 180 participants would provide approximately 0.80 power to detect medium effect sizes (*f* = 0.25) for a main effect of the group at post-training, after controlling for pre-training performance, using a series of between-group ANCOVAs. We aimed to power this to ensure sufficient power to address our primary research question: whether training gains are feature-specific or if they generalise across tasks. Following the study, a post-hoc power analysis based on our final sample size of 168 participants indicated a power of approximately 0.77 to detect medium effect sizes (*f* = 0.25) for group differences at post-training, after controlling for pre-training performance, using the same statistical approach.

## Results

Given the complexity of the design, we have included minimal descriptive statistics and focus on the primary inferential analyses of interest. However, full summary statistics broken down by different factor combinations are provided in the Supplemental Material.

### Transfer effects

**Figure 2.**
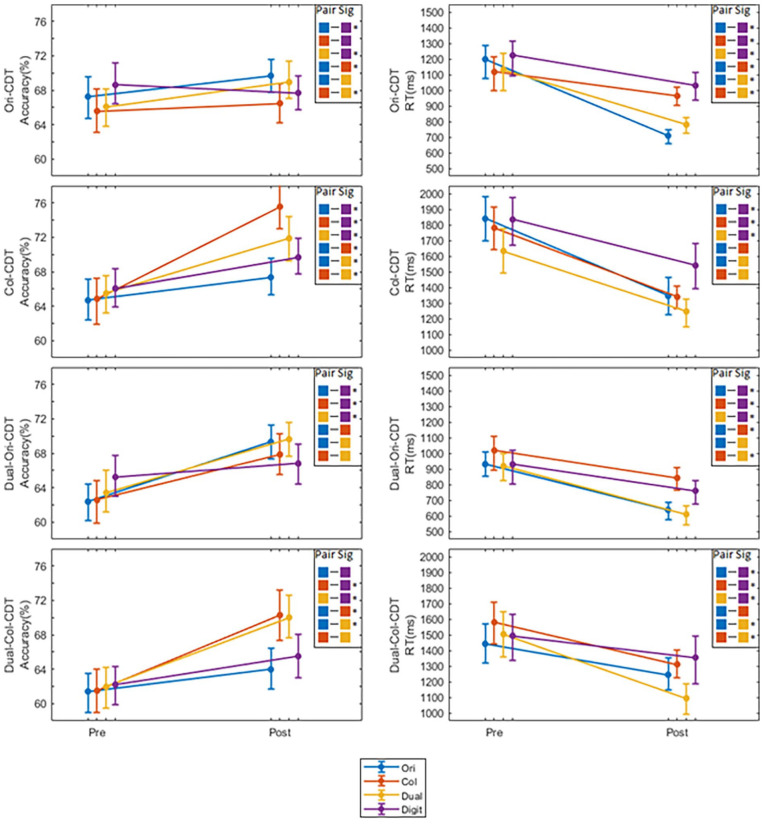
Mean accuracies and reaction times pre- and post-training for each group on each CDT task. *Note*. *p < .05. **p < .01. ***p < .001 (Group-wise holm-corrected). Significant group differences are shown at post-training after controlling for pre-training performance (Tables 1 and 2). Error bars show the 95% confidence interval about the mean. CDT = change-detection task.

To investigate whether the groups show differential transfer patterns with respect to either accuracy or reaction time, we first conducted a 4 (group) × 3 (set size) × 2 (cue type) ANCOVA for each of the CDT task conditions (Orientation, Colour, Dual-Orientation, and Dual-Colour), to test for any main effects of group, set size or cue type, and their interactions, whilst co-varying for pre-training performance. To test for differences in transfer to the Digit-Span task, we also performed a one-way ANCOVA to test for any group differences in span length, whilst co-varying for pre-training performance. The full results of these analyses, along with any other post-hoc follow-ups, are provided in the Supplemental Material. However, given that there were no group-by-set size, nor group-by-cue-type interactions, and for purposes of brevity, here we just report the main effects of group and associated post-hoc contrasts, at the task level (see Tables 1 and 2; Figure 2). In essence, the ANCOVAs revealed that the training effects do not interact with either set size or retro-cue/no cue.

**Table 1.** Pairwise group comparisons of the whole task mean accuracy differences adjusted for baseline performance.

Task	Group contrast	Post-training accuracy (%)/span difference	*t*-test			
			*df*	*t*	*p*	*d*
Ori-CDT	Ori-Digit	2.45	84	3.024	.013*	0.166
	Col-Digit	−0.22	83	0.272	1.000	0.015
	Dual-Digit	2.08	83	2.547	.033*	0.141
	Ori-Col	2.67	81	3.240	<.010**	0.184
	Ori-Dual	0.37	81	0.447	1.000	0.025
	Col-Dual	−2.30	80	2.780	.022*	0.159
Col-CDT	Ori-Digit	−1.95	84	2.007	.045*	0.140
	Col-Digit	6.22	83	6.344	<.001***	0.433
	Dual-Digit	2.36	83	2.405	.032*	0.163
	Ori-Col	8.17	81	−8.251	<.001***	0.576
	Ori-Dual	−4.31	81	4.351	<.001***	0.301
	Col-Dual	3.86	80	3.874	<.001***	0.262
Dual-Ori-CDT	Ori-Digit	3.60	84	4.491	<.001***	0.271
	Col-Digit	2.06	83	2.559	.040*	0.149
	Dual-Digit	3.53	83	4.379	<.001***	0.256
	Ori-Col	1.54	81	1.892	.176	0.123
	Ori-Dual	0.08	81	0.094	.924	0.006
	Col-Dual	−1.46	80	1.786	.176	0.122
Dual-Col-CDT	Ori-Digit	−1.23	84	1.351	.354	0.095
	Col-Digit	5.07	83	5.542	<.001***	0.366
	Dual-Digit	4.60	83	5.031	<.001***	0.334
	Ori-Col	−6.29	81	6.809	<.001***	0.470
	Ori-Dual	−5.83	81	6.301	<.001***	0.438
	Col-Dual	−0.47	80	0.503	.614	0.032
Digit-Span	Ori-Digit	−2.87	84	8.705	<.001***	1.413
	Col-Digit	−2.34	83	7.043	<.001***	1.174
	Dual-Digit	−2.54	83	7.613	<.001***	1.303
	Ori-Col	0.54	81	1.605	.330	0.329
	Ori-Dual	0.33	81	0.991	.646	0.211
	Col-Dual	0.20	80	0.599	.646	0.133

*Note*. CDT = change-detection task; Ori-CDT = Orientation Change-Detection Task; Col-CDT = Colour Change-Detection Task

* *p* < .05. **p < .01. ***p < .001 (holm-corrected).

**Table 2.** Pairwise group comparisons of the whole task mean reaction time differences adjusted for baseline performance.

Task	Group contrast	Post-training reaction time difference (ms)	Between-group *t*-test			
			*df*	*t*	*p*	*d*
Ori-CDT	Ori-Digit	−313.97	84	18.285	<.001***	1.270
	Col-Digit	−37.54	83	2.164	.030*	0.140
	Dual-Digit	−224.64	83	12.962	<.001***	0.882
	Ori-Col	−276.42	81	15.781	<.001***	1.471
	Ori-Dual	−89.33	81	5.104	<.001***	0.533
	Col-Dual	187.09	80	10.644	<.001***	0.948
Col-CDT	Ori-Digit	−197.28	84	6.321	<.001***	0.405
	Col-Digit	−184.19	83	5.861	<.001***	0.414
	Dual-Digit	−225.84	83	7.123	<.001***	0.502
	Ori-Col	−13.09	81	0.411	.747	0.036
	Ori-Dual	28.55	81	0.890	.747	0.078
	Col-Dual	41.65	80	1.296	.584	0.138
Dual-Ori-CDT	Ori-Digit	−123.68	84	8.184	<.001***	0.513
	Col-Digit	38.97	83	2.551	.021*	0.144
	Dual-Digit	−146.05	83	9.604	<.001***	0.513
	Ori-Col	−162.65	81	10.530	<.001***	0.718
	Ori-Dual	22.37	81	1.454	.146	0.113
	Col-Dual	185.03	80	11.894	<.001***	0.797
Dual-Col-CDT	Ori-Digit	−83.82	84	3.059	<.010**	0.177
	Col-Digit	−88.68	83	3.212	<.010**	0.190
	Dual-Digit	−266.45	83	9.671	<.001***	0.579
	Ori-Col	−4.86	81	0.173	.862	0.013
	Ori-Dual	182.63	81	6.547	<.001***	0.529
	Col-Dual	177.77	80	6.332	<.001***	0.528

*Note*. CDT = change-detection task; Ori-CDT = Orientation Change-Detection Task; Col-CDT = Colour Change-Detection Task.

* *p* < .05. **p < .01. ***p < .001 (holm-corrected).

### Accuracy

There was a significant main effect of group on post-training accuracy, whilst co-varying for pre-training performance, for all of the tasks: Ori-CDT (*F*(3,983) = 5.703, *p* < .001, $\eta_{p}^{2}$ = .017); Col-CDT (*F*(3,983) = 25.239, *p* < .001, $\eta_{p}^{2}$ = .071); Dual-Ori-CDT (*F*(3,983) = 8.876, *p* < .001, $\eta_{p}^{2}$ = .026); Dual-Col-CDT (*F*(3,983) = 23.983, *p* < .001, $\eta_{p}^{2}$ = .068); and Digit-Span (*F*(3,983) = 31.983, *p* < .001, $\eta_{p}^{2}$ = .068). To follow up on these main effects of group, post-hoc *t*-tests were conducted on the adjusted (for pre-training performance) post-training accuracy scores to establish precisely which group contrasts were significant (Table 1). The significant effects of group differences are summarised below.

### Ori-CDT training versus Digit-Span training (control)

Following training, the Ori-CDT training group had greater accuracy relative to the Digit-Span training group on the Ori-CDT and the Dual-Ori-CDT tasks. Conversely, the Digit-Span training group had greater accuracy relative to the Ori-CDT group on the Col-CDT and Digit-Span tasks. In other words, training on the Ori-CDT task provides benefits on that task, either alone or in the dual-task context, but appears to make participants significantly worse at the colour variant of the same task (Col-CDT), relative to the active control group.

### Col-CDT training versus Digit-Span training (control)

Following training, the Col-CDT training group had greater accuracy relative to the Digit-Span training group on the Col-CDT, Dual-Ori-CDT, and Dual-Col-CDT. Conversely, the Digit-Span training group had greater accuracy relative to the Col-CDT training on the Digit-Span task. To summarise, unlike training on the Ori-CDT, Col-CDT benefits *both* variants of the change-detection paradigm in the dual context, again relative to the active control condition.

### Dual-CDT training versus Digit-Span training (control)

Following training, the Dual-CDT training group had greater accuracy relative to the Digit-Span training group on the Ori-CDT, Col-CDT, Dual-Ori-CDT and Dual-Col-CDT tasks. This would suggest that change-detection training in the dual-task context yields benefits for both variants of the change-detection paradigm, regardless of whether they are presented alone or in the dual-task context, relative to the active control group. Conversely, the Digit-Span training group had greater accuracy relative to the Dual-CDT training on the Digit-Span task.

### Ori-CDT training versus Col-CDT training

Following training, the Ori-CDT training group had greater accuracy relative to the Col-CDT training group on the Ori-CDT task. Conversely, the Col-CDT group had greater accuracy relative to the Ori-CDT training group on the Col-CDT, and Dual-Col-CDT tasks. This is the clearest evidence that the Col-CDT training provides a performance boost that transfers across contexts (both stand-alone CDT and within a dual-task context), relative to the Ori-CDT training, which only appears to boost performance on that task in its stand-alone context.

### Dual-CDT training, versus the task-specific variants

As we might expect, the Dual-CDT training yields benefits over the task-specific versions of the training, on whichever variant they do not train. For instance, Dual-CDT training participants are significantly better than the Ori-CDT training group on the Col-CDT task, and vice versa. However, the Col-CDT group does get an extra boost to performance on that task, relative to the Dual-CDT group, suggesting that there is some additional benefit for performance on that variant by training solely on it.

### Reaction time

There was a significant main effect of group on post-training RT, whilst co-varying for pre-training performance, for all of the CDT tasks: Ori-CDT (*F*(3,983) = 150.370, *p* < .001, $\eta_{p}^{2}$ = .314); Col-CDT (*F*(3,983) = 21.614, *p* < .001, $\eta_{p}^{2}$ = .061); Dual-Ori-CDT (*F*(3,983) = 69.599, *p* < .001, $\eta_{p}^{2}$ = .175); and Dual-Col-CDT (*F*(3,983) = 32.744, *p* < .001, $\eta_{p}^{2}$ = .090). The Digit-Span task was excluded from any RT analysis because there was no speeded component to it. To follow up on these main effects of the group, post-hoc *t*-tests were conducted on the adjusted (for pre-training performance) post-training RT scores to establish precisely which group contrasts were significant (Table 2). The significant effects of group differences are summarised below.

### CDT training versus Digit-Span training (control)

With only one exception, training on any version of the CDT paradigm made participants significantly faster on *any other* version of the CDT paradigm, relative to the active control group. In other words, CDT training appears to provide very general RT benefits relative to active control.

### Ori-CDT training versus Col-CDT training

Following training, the Ori-CDT training group was faster relative to the Col-CDT training group on the Ori-CDT and Dual-Ori-CDT tasks. In other words, the Ori-CDT task provides a specific benefit to that task regardless of the dual-task context, relative to the other variant of the change-detection paradigm. The reverse is not true; however, training on the Col-CDT only appears to confer an RT benefit relative to the Ori-CDT in the Col-CDT task but not in the dual-task context.

### Dual-CDT training versus the task-specific variants

Relative to the Col-CDT, the Dual-CDT training makes participants significantly better at all other types of change-detection paradigms save for the stand-alone Col-CDT paradigm on which those participants trained. By contrast, those trained on the Ori-CDT alone get a significant boost on the stand-alone variant of that task, relative to those trained in the dual-task condition. However, the dual-task trained individuals get a significant boost on the colour change-detection paradigm in the dual-task context, relative to those trained on Ori-CDT alone.

### Psychometric functions

**Figure 3.**
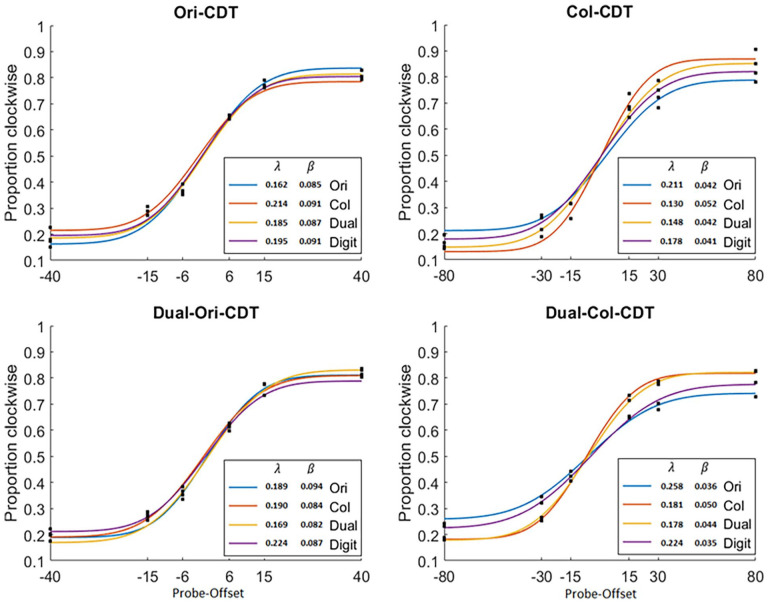
Cumulative Gaussian functions fit each of the CDT tasks for each group across trials. *Note*. CDT = change-detection task.

In addition to comparing groups on mean accuracy and RT, we also wanted to investigate whether group differences in training outcomes on the CDT tasks were driven by an increase in the *number of*, or the *quality of*, item representations held in memory. To do so, we fit cumulative Gaussian curve models to the distributions of clockwise responses over the range of target-stimulus offsets for each group, on each task (collapsed across set sizes 2 and 4, and cue type), according to the methods outlined in Murray et al. (2013), using the Palamedes toolbox (Prins & Kingdom, 2018). For this set of analyses, we omitted the set size 8 trials due to the noise caused by high levels of variability on these trials. Each of these models provided two key parameters of interest: λ (Lambda) – the asymptote of the curve, providing an estimate of the probability that a probe item was represented in memory (lower numbers indicate a higher probability of recall) and β (Beta) – the slope of the curve, providing an estimate of the precision of a probe item representation (higher numbers indicate greater precision). The models and the corresponding parameters of interest are shown in Figure 3 below.

The functions were fit to the group as a whole post-training, combining across trials from all participants. This was to try and maximise the number of trials and improve the overall fit of the model (See section ‘Discussion’; Murray et al., 2013; Prins & Kingdom, 2018). The statistical comparisons between groups were then conducted by using 500 bootstraps to create confidence intervals for each group. These could then be used to compare the groups statistically (whilst correcting for multiple comparisons; Table 3).

**Table 3.** Pairwise group comparisons of the whole task differences on key psychometric parameters.

Task	Group contrast	Differences			
		*B*	*p*	λ	*p*
Ori-CDT	Ori-Digit	−0.006	1.000	−.033	.240
	Col-Digit	−0.001	.832	.018	.504
	Dual-Digit	−0.005	1.000	−.012	.416
	Ori-Col	−0.006	1.000	−.051	.012*
	Ori-Dual	−0.001	1.000	−.021	.552
	Col-Dual	−0.004	1.000	.030	.552
Col-CDT	Ori-Digit	0.001	.908	.032	.276
	Col-Digit	0.011	.160	−.047	.040*
	Dual-Digit	0.001	1.000	−.032	.276
	Ori-Col	0.011	.272	.079	<.001***
	Ori-Dual	−0.001	1.000	.064	.048*
	Col-Dual	0.010	1.200	−.014	.308
Dual-Ori-CDT	Ori-Digit	0.001	.960	−.023	.800
	Col-Digit	−0.004	.780	−.022	.800
	Dual-Digit	−0.006	.960	−.044	.396
	Ori-Col	0.011	.740	−.001	.500
	Ori-Dual	0.013	.372	.021	.736
	Col-Dual	0.002	.728	.022	.576
Dual-Col-CDT	Ori-Digit	0.001	.430	.035	.240
	Col-Digit	0.015	.060	−.042	.264
	Dual-Digit	0.009	.216	−.047	.264
	Ori-Col	−0.014	.036*	.076	.024*
	Ori-Dual	−0.008	.216	.081	.024*
	Col-Dual	−0.006	.300	.005	.432

*Note*. CDT = change-detection task; Ori-CDT = Orientation Change-Detection Task; Col-CDT = Colour Change-Detection Task.

* *p* < .05. ***p < .001 (Holm-corrected).

The Ori-CDT group showed a significantly higher probability of recall (Lambda) relative to the Col-CDT group when performing the Ori-CDT assessment. The Col-CDT group showed a significantly higher probability of recall relative to both the control group and the Ori-CDT group when performing the Col-CDT assessment. The Dual-CDT group showed a significantly higher probability of recall relative to the Ori-CDT group when performing the Col-CDT assessment. The Col-CDT group showed a significantly higher probability of recall and greater precision relative to the Ori-CDT group when performing the Dual-Col-CDT assessment. Finally, the Dual-CDT group showed a significantly higher probability of recall relative to the Ori-CDT group when performing the Dual-Col-CDT assessment. In other words, the majority of the significant training effects can be found on the asymptote of the curve, and not the slope.

### Correlations pre- and post- training

To explore further the relationships between tasks and how these might change as a function of training, we examined the correlations at pre-assessment, post-assessment, and the difference between the two (Table 4). To establish whether correlations changed significantly following training, we used a permutation method wherein we randomly sampled (*n* = 2,000) the pre- and post-assessment task performances for each group and calculated the pairwise changes in the correlation coefficients to estimate a distribution and produce *p*-values. The only significant difference was an increased correlation between the Col-CDT versus Dual-Col-CDT for the Dual-CDT training group.

**Table 4.** Pairwise comparisons of the changes in correlations between change-detection tasks following training within each group.

Task pair	Group	Correlations			
		Pre	Post	Difference	*p*
Ori versus Col	Ori-CDT	.474	.555	.081	.261
	Col-CDT	.614	.523	−.091	.285
	Dual-CDT	.514	.636	.123	.158
	Digit-Span	.541	.463	−.077	.260
Ori versus Dual-Ori	Ori-CDT	.744	.809	.065	.247
	Col-CDT	.814	.818	.004	.470
	Dual-CDT	.676	.680	.004	.480
	Digit-Span	.685	.806	.121	.075
Col versus Dual-Ori	Ori-CDT	.320	.465	.145	.170
	Col-CDT	.746	.526	−.220	.056
	Dual-CDT	.445	.442	−.003	.454
	Digit-Span	.624	.582	−.041	.352
Ori versus Dual-Col	Ori-CDT	.490	.605	.114	.224
	Col-CDT	.594	.683	.090	.230
	Dual-CDT	.535	.644	.108	.206
	Digit-Span	.474	.572	.097	.254
Col versus Dual-Col	Ori-CDT	.715	.713	−.002	.487
	Col-CDT	.755	.806	.051	.267
	Dual-CDT	.521	.857	.336	<.001***
	Digit-Span	.737	.624	−.112	.121
Dual-Ori versus Dual-Col	Ori-CDT	.570	.655	.084	.271
	Col-CDT	.801	.694	−.107	.078
	Dual-CDT	.592	.549	−.043	.321
	Digit-Span	.643	.753	.111	.181

*Note*. CDT = change-detection task; Ori-CDT = Orientation Change-Detection Task; Col-CDT = Colour Change-Detection Task.

*** *p* < .001.

We also compared the differences between groups (Table 5). To establish whether the between-group contrasts for correlation differences following training were significant, we used the permuted pre- and post-samples from the above analyses to form a single distribution of the differences and estimated *p*-values according to the proportion of the distribution above or below 0 (two-tailed). There was a significant difference between the Ori-CDT and Col-CDT training groups on the Col-CDT versus the Dual-Ori-CDT task pair, with the two tasks becoming relatively less correlated for the Col-CDT group. There were also significant differences between Dual-CDT and all the other training groups on the Col-CDT versus Dual-Col-CDT task pair, with the correlation between these two tasks becoming relatively increased in each case.

**Table 5.** Group contrasts for the pairwise changes in correlations between the change-detection tasks following training.

Task pair	Group contrast	Difference in *r*-change	*p*
Ori versus Col	Ori-Digit	.158	.201
	Col-Digit	−.013	.464
	Dual-Digit	.200	.121
	Ori-Col	.172	.199
	Ori-Dual	−.042	.384
	Col-Dual	−.214	.146
Ori versus Dual-Ori	Ori-Digit	−.055	.293
	Col-Digit	−.116	.157
	Dual-Digit	−.116	.201
	Ori-Col	.061	.336
	Ori-Dual	.061	.333
	Col-Dual	−.001	.463
Col versus Dual-Ori	Ori-Digit	.186	.147
	Col-Digit	−.179	.184
	Dual-Digit	.039	.429
	Ori-Col	.365	.046*
	Ori-Dual	.147	.224
	Col-Dual	−.218	.181
Ori versus Dual-Col	Ori-Digit	.017	.499
	Col-Digit	−.008	.481
	Dual-Digit	.011	.480
	Ori-Col	.025	.486
	Ori-Dual	.006	.490
	Col-Dual	−.019	.475
Col versus Dual-Col	Ori-Digit	.110	.197
	Col-Digit	.163	.086
	Dual-Digit	.449	<.001***
	Ori-Col	−.053	.350
	Ori-Dual	−.338	.011*
	Col-Dual	−.285	.017*
Dual-Ori versus Dual-Col	Ori-Digit	−.027	.439
	Col-Digit	−.218	.054
	Dual-Digit	−.154	.163
	Ori-Col	.191	.119
	Ori-Dual	.127	.220
	Col-Dual	−.064	.380

*Note*. CDT = change-detection task.

* *p* < .05. ***p < .001 (Holm-corrected).

## Discussion

This online study examined transfer patterns following training using a set of nested CDTs. This design allowed us to ask specific, theoretically motivated questions about transfer, its boundary conditions, and potential underlying mechanisms. Specifically, we asked the following questions: (a) Does training lead to the acquisition of skills that enhance the number of items stored in memory, the precision of those items, or both? (b) Does training interact with the spatial allocation of attention? (c) Are the skills acquired during training in the single judgement tasks bound to their specific judgement types of colour and orientation, or do they transfer to one another? (d) Do the skills acquired during training in the single task conditions transfer ‘up’ the task hierarchy to a dual judgement (Orientation and Colour) task and vice versa?

The broad pattern of data showed both accuracy and RT improvements following training, with each assessment task showing an effect of group. That is, for each task, the degree of improvement shown, relative to the pre-training baseline, was moderated by the kind of training participants had undertaken. However, there were no significant interactions with set size or cue type (no-cue or retro-cue trials), so for all following analyses we collapsed across these factors. There were a number of interesting group-specific training effects. For example each CDT training group made significantly greater on-task gains, relative to the active control group. Participants who underwent colour-CDT or orientation-CDT training gained significantly on their respective assessment tasks, but there was no significant transfer between them. On the contrary, the orientation training group was *significantly worse* than the control group at the colour-CDT assessment. Rather than recapitulate each result here, because there are so many, we will group the key results around the four research questions we attempted to answer with this study.

### Does training lead to the acquisition of skills that enhance the number of items stored in memory, the precision of those items or both?

Previous research has shown attentional and/or experiential modulation of both the quantity (Buschkuehl et al., 2017; Griffin & Nobre, 2003; Heuer & Schubo, 2016; Moriya, 2019; Norris et al., 2020) and quality (Bays et al., 2009; Heuer & Schubo, 2016; Marshall & Bays, 2013; Moriya, 2019) of VWM representations. As such, we predicted that training would enhance task performance with respect to both the number of item representations held in memory and the precision of those item representations. We tested this in two ways: (a) varying the number of items (set size) in the memory array and (b) varying the degree of offset for both the colour and the orientation of the probe stimulus relative to its counterpart in the memory array.

If training primarily enhanced capacity, then we would expect the CDT training groups to outperform the control group disproportionality on the higher set sizes compared to the lower set sizes. However, despite training being aimed at increasing capacity, there were no significant group-by-set size interactions on any of the tasks, suggesting that by this measure at least, capacity has not been increased. Varying the probe offsets allowed us to fit psychometric functions to the data and derive two key parameters of interest, for each group on each of the tasks. The first indicates sensitivity to changes in offset (Beta, i.e. quality of the representation), and the other indicates the threshold for the number of items held (Lambda). First, with respect to the Beta parameter, none of the CDT training groups showed greater sensitivity to change relative to controls either on or across tasks. Second, with respect to the Lambda parameter, the Col-CDT training group showed a significantly higher capacity threshold relative to controls on-task, suggesting that training on the Col-CDT task does in fact engender skills related to capacity. However, neither the Ori-CDT nor Dual-CDT training groups showed significantly higher capacity thresholds, on- or across tasks, relative to controls.

Further to the comparison with controls, there were some specific effects for these parameters when comparing the CDT training groups. For example on the Col-CDT task, the Col-CDT training group showed both enhanced precision and an enhanced capacity threshold relative to the Ori-CDT group. Relatedly, both the Col-CDT and Dual-CDT training groups showed an enhanced capacity threshold relative to the Ori-CDT group. Conversely, the Ori-CDT training group showed a greater capacity threshold on the Ori-CDT task compared to the Col-CDT. These findings mirror those from the whole task accuracy comparisons and suggest that there are some specific training effects associated with the judgement type trained. Moreover, given that these effects only come out between training groups and not relative to controls, indicates that not only are some of the skills acquired task-specific but also that there may be some skills acquired that are detrimental outside of the original training context. In other words, training on the Orientation-CDT may make your colour CDT slightly worse, at least relative to the control group.

Taken together, the above findings suggest that there are some task-specific effects pertaining to both quantity and quality of item representations. However, these effects are patchy at best, and reconciling them becomes tricky when integrating them with the far more robust basic accuracy effects between groups. One explanation for why we might find performance differences between the CDT training groups and controls on simple accuracy measures, but not in the precision or capacity estimates from the psychometric modelling, is that training affects *both* the quality and quantity of item representations. When these are essentially aggregated with crude overall accuracy measures, they are more robustly detected, whereas when they are segregated into modelling components, the effects wash out when we control for multiple comparisons. Relatedly, we were only able to fit the psychometric functions in a coarse manner across set sizes at post-training. This means that any effects are likely dulled by virtue of having to exclude the noisy supra-threshold set size 8 trials. This is further amplified by not having access to the full data due to the server error. With more data, the same analysis may allow us to disentangle effects on precision versus capacity more convincingly. Moreover, comparing across groups post-training does not account for any potential pre-training differences, as in the other analyses.

There is an ongoing discussion about whether training and transfer effects in WM tasks reflect enhancements in capacity or efficiency (e.g. see von Bastian et al., 2022). The capacity-efficiency model suggests that cognitive training can induce transfer through two primary pathways: by expanding cognitive capacity or by improving efficiency in utilising the existing capacity (though not mutually exclusive). Evidence suggests that the training and transfer effects found in cognitive training studies to date likely reflect the latter – that is, efficiency – rather than the former. This is largely implied by the observed transfer patterns – near transfer to similar tasks where efficiencies (automatisations, routines, strategies, etc.) can be effectively applied, and a lack of far transfer to tasks for which the trained strategy does not apply. The findings in this study align with these ideas. The limited transfer effects observed and the lack of a robust set size effect suggest that performance improvements are more likely due to acquired context-specific efficiencies rather than a general WM capacity increase. It is, however, worth noting that task-specific capacity could still increase independently of general WM limits, potentially as a result of resources being freed through such efficiencies.

To summarise this first question: there is no compelling evidence that would allow us to conclude that CDT training significantly impacts capacity or precision selectively. There are no interactions with set size, and relative to controls, only one significant improvement to the asymptote of the psychometric functions. There are no significant improvements in the slope of the psychometric functions, relative to controls.

### Does training interact with the spatial allocation of attention?

Top-down attentional modulation has been shown to impact both the encoding and maintenance of CDT item representations (Astle et al., 2012; Griffin & Nobre, 2003; Posner, 1980). Prior to data collection, we tentatively predicted that training would disproportionately affect processes associated with the encoding/early maintenance (i.e. those prior to cue onset) phase of the trial due to the relatively long presentation times used here, which may leave more room for strategically modifying encoding efficiency (Gaspar et al., 2013; Vogel et al., 2006), and evidence suggesting an increased value of placing cues early on at encoding/maintenance (Astle et al., 2012; Griffin & Nobre, 2003). An alternative possibility is that training boosts the allocation of spatial attention during maintenance.

To distinguish these two possibilities, we included a retro-cue on half of the assessment CDT task trials, halfway (500 ms) through the maintenance phase of the trial to indicate the position of the upcoming probe stimulus. The idea being that if training disproportionately affected processes earlier on in the trials, then we would expect any training effects to persist regardless of the cue, relative to the control group. In other words, the cue type should not interact with the training group. Alternatively, if the training affected later processes, then the training gains might be negated by the cue in on-cue trials, and everyone can orient their top-down attention regardless of what training they have had. In other words, we would get an interaction between group and cue type – the training gains are only present on no-cue trials.

In line with previous findings, there was a main effect of cue type for both accuracy and RT, with cue trials both enhancing accuracy and decreasing RT (Astle et al., 2012; Griffin & Nobre, 2003; Souza & Oberauer, 2016), on all tasks except for accuracy on the Ori-CDT task where the effect was null. However, crucially, there were no group-by-cue-type interactions, indicating that whatever was learnt during training was unaffected by the presence of the cue. One possible interpretation is simply that whatever is gained during training has nothing to do with the spatial orientation of attention during the maintenance phase. If it did, then we would expect it to interact with the cueing effect. This may suggest that whatever is enhanced by the training reflects some other mechanism, either early at encoding, or late during retrieval, but it does not pertain to the allocation of attention during VWM maintenance.

### Are the skills acquired during training in the single judgement tasks bound to their specific judgement types of colour and orientation, or do they transfer to one another?

Prior to data collection, we predicted that skill gains associated with *enhancing the quality* of memory representations would be judgement specific, but that any gains stemming from *enhanced capacity* would be transferable. The first half of this prediction was based upon increased saliency for the trained features. There is evidence suggesting that memory recall for within-object features can fail independently in continuous response paradigms (Bays et al., 2011; Fougnie & Alvarez, 2011). The second half of this prediction was based on evidence suggesting that maintaining a greater number of items requires within-object features to be bound as integrated items (Alvarez & Cavanagh, 2004; Bays & Husain, 2008; Bays et al., 2009; Luck & Vogel, 2013; Luria & Vogel 2011; Oberauer & Hein, 2012). Moreover, a prior study (Norris et al., 2019) showed a transfer between orientation and colour judgement types in a CDT paradigm following colour-CDT training in terms of overall capacity.

However, as previously discussed, we had difficulty parsing quantity versus quality training effects. Despite a tentative indication that some effects may be more driven by skills pertaining to quantity (i.e. asymptote), we now consider this question across both of these at the whole task level with respect to both accuracy and RT. In the accuracy data (i.e. when comparing mean accuracy across groups), the data speak strongly to the gains being feature specific. Being trained to remember an increased number of items in terms of their orientation does not improve your memory for their colour and vice versa. Thus, we did not replicate the findings of Norris et al. (2019). On the contrary, there was instead evidence for ‘negative transfer’ – training on the orientation variant makes you worse at the colour variant, at least relative to the controls. There was a hint of this in the psychometric function data also. One possibility is that this is driven by some inhibitory process; training on the orientation variant may actively encourage participants to suppress the interfering colour information that is bound within the stimulus, or that the orientation information is biased to such an extent that it becomes interfering when the colour information is relevant. A key difference between this study and that of Norris et al. (2019) is that we used feature-bound items, such that the perceptual characteristics of the memoranda were matched across all CDT tasks. In contrast, Norris et al. (2019) used items that contained colour information or orientation information, but never both. Another difference is that we varied the offset of the probe stimulus, whereas Norris et al. (2019) left theirs constant and at an easily discriminable level. As mentioned above, one possibility is that varying the degree of discriminability in this study encouraged a very feature-specific strategy, which may have prevented transfer. These factors may explain the differences between the two studies.

The RT data show far more widespread improvements. Training on any variant of the CDT makes you faster at all other variants of the CDT task. There are also some additional improvements more specific to the Orientation-CDT and Dual-CDT group. Both groups are faster than the Col-CDT group when orientation judgements are required in either a single judgement or dual judgement context. Similarly, the Dual-CDT group is quicker on colour judgements than other groups for colour judgements in the dual condition. These effects are hard to interpret because the improved speed at a task could result from any number of processes. However, one likely possibility is that CDT training enhances retrieval speeds and/or motor responses. The response required in all the CDT variants is to use a colour wheel or orientation wheel to decide whether the stimulus had rotated clockwise or anticlockwise. This is quite a bizarre thing for participants to do, and a strong possibility is that this response process itself is trainable. Because all CDT variants use this response method, getting faster will likely transfer across tasks. This may be why the RT data appear to show such generic transfer effects.

### Do the skills acquired during training in the single task conditions transfer ‘up’ the task hierarchy to a dual judgement (Orientation and Colour) task and vice versa?

Whilst we did not make any specific predictions about the directionality of transfer at pre-registration, given the findings from Rennie et al. (2021), it seems reasonable now to assume that we would observe transfer ‘down’ the hierarchy, but not transfer ‘up’. That is, we might observe improvements in the simpler CDT variants from having trained on the dual version, but not vice versa. The accuracy data demonstrate that in some cases, transfer along the hierarchy can be bidirectional. Those who trained on the dual variant of the CDT also gained significantly on the simpler feature-specific versions of the tasks, relative to controls. Also, whilst there may not be significant transfer between the single orientation and colour variants, colour-CDT trainees do get better at the orientation elements of the dual CDT, relative to controls. Meanwhile, the orientation-CDT trainees do not show this generalisation. They only improve on the orientation elements of the dual CDT (again with a similar effect size to the dual trainees). It thus seems from the accuracy data that training on either colour or orientation-CDT boosts performance wherever you encounter that stimulus type, and to roughly the same extent as those who have trained on the dual version (in terms of effect size). Likewise, training on the dual version makes you better on either of the simpler versions, again with a similar effect size to those who train selectively on those respective variants. Interestingly, however, colour-CDT trainees show transfer to orientation only within the context of the dual CDT. One possible explanation is that the gains for the colour variant are simply much bigger. If the colour variant becomes much easier for those who have encountered it during training, then it may free up resources for those participants to allocate to the orientation feature of the dual task. In essence, the training gains themselves are somewhat asymmetric, and this may explain the apparent asymmetry of transfer.

### Correlational relationships following training

In both Rennie et al. (2020, 2021), there were several changes in pairwise task correlations within-group following training. By contrast, here only one task pair changed within a single group: following Dual-CDT training, there was a substantial increase in association between the colour-CDT task and the dual-colour CDT, perhaps indicating that they now recruit more common processes. However, it is curious that we do not see the same gain in association with colour-CDT training group. One possibility is that the additional executive demands of the dual condition interfere with the colour-CDT groups’ usual on-task processes – in essence because these participants have not encountered the dual condition, other than in the assessments, it still involves additional processes and thus the correlation between the colour-CDT variants (alone versus in the dual context) is smaller.

One shortcoming of Rennie et al. (2021) was a lack of between-group comparisons for these correlation effects. However, here we also compared these changes between groups. In accordance with the above effect, the Dual-CDT training group sees a greater strengthening of the association between the Col-CDT task and the Dual-Col-CDT task, relative to the other groups. Further to this, when contrasted against one another, we see a weakening of association between the Col-CDT and Dual-Ori-CDT tasks for the Col-CDT training group relative to the Ori-CDT training group, for whom we see a slight strengthening of association. This is perhaps further evidence for feature-specific training gains, which drive strong correlations between variants that share the feature, and for some mild interference caused by judgement types other than those trained in the single judgement conditions.

### Limitations

There are several limitations to the current study that are important to consider when interpreting the findings. First, we were missing some of the assessment data due to a technical error with the JATOS server. In practice, this means that the analyses are slightly underpowered relative to the original design (we are missing 10% of the participants’ assessment data). The original power calculation can be sourced from the pre-registration and can be found here (https://osf.io/nb5m7). Based on our actual sample size, we have 0.78 power to detect the predicted effect sizes. Second, the psychometric modelling excludes the set size 8 trials, which were incredibly noisy. A more optimal design would have been to avoid these trials altogether, but we had worried we would get ceiling effects at set size 4 following training. Third, ideally, we would have more trials such that we could fit good psychometric functions for individual participants. In the current analysis, we fit the functions at a group level, with bootstraps used for group-wise comparisons, but with more trials, it may be possible to fit them per subject.

### Conclusions

By training VWM using three variants of a CDT task, alongside an active control group, we were able to answer a number of key questions about patterns of transfer. First, training on either a simple variant of the CDT does not transfer to the other. On the contrary, training on the orientation version may make you somewhat worse at the colour version. Second, transfer patterns are bidirectional within the nested hierarchy. Training on the dual version of the CDT makes participants almost as good at the simple versions as if they had trained selectively on just those tasks. Likewise, training on the simple versions makes participants better at those elements of the more complex task. In the case of colour-CDT trainees, they show improvements in the orientation elements of the dual-CDT task, suggesting between-variant transfer only in the context of the dual task. Third, whatever is being trained in CDT is unlikely to be the spatial allocation of attention during the maintenance period. There are no interactions between training type and cue type. Whatever is enhanced by the training likely happens either pre-cue, such as during encoding, or during retrieval. Finally, we were not able to conclude as to whether training enhances capacity or precision because the results from the psychometric function analysis do not clearly support one or the other.

## Supplemental Material

sj-docx-1-qjp-10.1177_17470218251334370 – Supplemental material for Up, down, and all around? Deciphering the boundary conditions for training-induced transfer effects within a set of hierarchically nested tasks Supplemental material, sj-docx-1-qjp-10.1177_17470218251334370 for Up, down, and all around? Deciphering the boundary conditions for training-induced transfer effects within a set of hierarchically nested tasks by Joseph Rennie and Duncan E Astle in Quarterly Journal of Experimental Psychology
