# Supplementary material for: Up, down, and all around? Deciphering the boundary conditions for training-induced transfer effects within a set of hierarchically nested tasks
Source: Q J Exp Psychol (Hove). 2025 Apr 2;79(6):1406–28. doi: 10.1177/17470218251334370 (PMC13168620; doi:10.1177/17470218251334370)
Supplement: sj-docx-1-qjp-10.1177_17470218251334370 – Supplemental material for Up, down, and all around? Deciphering the boundary conditions for training-induced transfer effects within a set of hierarchically nested tasks [file sj-docx-1-qjp-10.1177_17470218251334370.docx]

Supplementary Material for:

**Up, down, and all around? Deciphering the boundary conditions for training induced transfer effects within a set of hierarchically nested tasks.**

Joseph Rennie & Duncan E. Astle

*Corresponding Author:

Dr Joseph Rennie,

Washington Singer Laboratories,

University of Exeter,

Perry Road,

Prince of Wales Road,

Exeter,

EX4 4QG,

UK

[J.P.Rennie@exeter.ac.uk](mailto:J.P.Rennie@exeter.ac.uk)

*Accuracy and reaction time summary statistics broken down by different factor combinations.*

The following pages contain summary statistics for Accuracy and Reaction time broken down by various factor combinations.

| Table S1. Descriptive statistics for the digit-span task pre and post. | | | | | | | |
| --- | --- | --- | --- | --- | --- | --- | --- |
|  | | Span length | | | | | |
| Task | Training group | Pre-training | | Post-training | | Difference | |
|  |  | M | SD | M | SD | M | SD |
| Digit-Span | Ori-CDT | 7.00 | 1.56 | 7.07 | 1.68 | 0.07 | 1.53 |
|  | Col-CDT | 6.85 | 1.40 | 7.51 | 1.56 | 0.66 | 1.42 |
|  | Dual-CDT | 6.41 | 1.24 | 7.02 | 1.45 | 0.60 | 1.24 |
|  | Digit-Span | 6.83 | 1.57 | 9.83 | 2.31 | 3.00 | 2.05 |
|  | | | | | | | |

Table S2. Summary statistics for change detection accuracy performance across set-sizes pre and post, split by cue-type.

Accuracy (%)

Task

Training group

Across cue-type Cue No-cue

Pre-training Post-training Difference Pre-training Post-training Difference Pre-training Post-training Difference

|  | M | SD | M | SD | M | SD | M | SD | M | SD | M | SD | M | SD | M | SD | M | SD |
| --- | --- | --- | --- | --- | --- | --- | --- | --- | --- | --- | --- | --- | --- | --- | --- | --- | --- | --- |
| Ori-CDT | 67.25 | 8.28 | 69.69 | 6.40 | 2.44 | 7.25 | 69.97 | 10.31 | 70.98 | 7.21 | 1.00 | 8.74 | 64.52 | 7.63 | 68.4 | 6.78 | 3.87 | 7.94 |
| Col-CDT  Ori-CDT  Dual-CDT | 65.57  66.08 | 8.32  7.27 | 66.48  68.94 | 7.94  6.9 | 0.91  2.86 | 7.01  7.18 | 68.44  70.22 | 10.5  9.47 | 67.48  70.26 | 8.79  8.43 | -0.97  0.04 | 8.61  9.60 | 62.70  61.95 | 7.66  6.17 | 65.47  67.63 | 8.02  6.87 | 2.78  5.68 | 8.08  6.97 |
| Digit-Span | 68.61 | 8.01 | 67.69 | 6.74 | -0.92 | 6.21 | 73.01 | 10.04 | 69.65 | 8.23 | -3.36 | 7.71 | 64.22 | 7.33 | 65.73 | 6.58 | 1.51 | 6.67 |
| Ori-CDT | 64.67 | 8.15 | 67.35 | 7.37 | 2.69 | 8.17 | 66.38 | 9.22 | 71.06 | 7.50 | 4.68 | 10.25 | 62.96 | 8.32 | 63.65 | 8.71 | 0.69 | 7.80 |
| Col-CDT | 64.85 | 8.83 | 75.57 | 9.68 | 10.72 | 10.93 | 67.07 | 9.80 | 78.70 | 9.59 | 11.63 | 11.11 | 62.63 | 8.99 | 72.44 | 10.34 | 9.81 | 11.63 |
| Col-CDT Dual-CDT | 65.5 | 7.06 | 71.88 | 8.68 | 6.38 | 7.72 | 69.00 | 8.69 | 75.11 | 9.46 | 6.11 | 9.07 | 62.00 | 7.72 | 68.65 | 9.24 | 6.64 | 9.78 |
| Digit-Span | 66.07 | 7.43 | 69.67 | 7.14 | 3.59 | 7.31 | 67.85 | 8.38 | 72.71 | 8.51 | 4.86 | 9.22 | 64.29 | 7.54 | 66.62 | 7.03 | 2.32 | 7.37 |
| Ori-CDT | 62.38 | 7.30 | 69.33 | 6.56 | 6.96 | 5.29 | 65.17 | 9.07 | 72.46 | 7.81 | 7.30 | 7.03 | 59.59 | 7.31 | 66.20 | 6.63 | 6.61 | 6.98 |
| Dual-Ori- Col-CDT | 62.56 | 7.94 | 67.86 | 7.8 | 5.31 | 5.82 | 65.33 | 9.81 | 71.09 | 8.97 | 5.76 | 7.85 | 59.78 | 7.52 | 64.63 | 7.88 | 4.85 | 6.07 |
| CDT Dual-CDT | 63.40 | 8.13 | 69.64 | 6.54 | 6.24 | 7.85 | 67.12 | 9.71 | 73.14 | 8.05 | 6.02 | 9.48 | 59.69 | 7.38 | 66.15 | 6.62 | 6.46 | 8.23 |
| Digit-Span | 65.24 | 7.80 | 66.80 | 7.88 | 1.57 | 6.72 | 69.19 | 10.26 | 71.19 | 9.96 | 2.00 | 8.73 | 61.28 | 6.50 | 62.42 | 7.23 | 1.14 | 7.62 |
| Ori-CDT | 61.41 | 7.38 | 63.97 | 8.06 | 2.56 | 7.31 | 63.14 | 9.05 | 67.11 | 9.11 | 3.97 | 9.01 | 59.68 | 7.33 | 60.82 | 8.34 | 1.15 | 8.74 |
| Dual-Col- Col-CDT | 61.50 | 8.42 | 70.29 | 9.58 | 8.80 | 7.63 | 63.87 | 9.73 | 74.21 | 9.52 | 10.34 | 8.69 | 59.12 | 8.09 | 66.37 | 10.39 | 7.25 | 8.67 |
| CDT Dual-CDT | 61.95 | 7.77 | 70.01 | 7.83 | 8.06 | 7.25 | 64.90 | 9.02 | 74.23 | 9.17 | 9.33 | 9.32 | 58.99 | 7.57 | 65.79 | 7.71 | 6.80 | 6.97 |
| Digit-Span | 62.21 | 7.68 | 65.51 | 8.58 | 3.30 | 7.12 | 64.56 | 10.06 | 68.37 | 10.13 | 3.81 | 9.10 | 59.85 | 7.01 | 62.65 | 7.89 | 2.80 | 7.89 |

| Table S3. Summary statistics for change detection reaction time performance across set sizes pre and post, split by cue-type. | | | | | | | | | | | | | | | | | | | |
| --- | --- | --- | --- | --- | --- | --- | --- | --- | --- | --- | --- | --- | --- | --- | --- | --- | --- | --- | --- |
| Reaction time (ms) | | | | | | | | | | | | | | | | | | | |
|  | Training group |  |  | Across cue-type | |  |  |  |  | Cue |  |  |  |  |  | No-cue | |  |  |
| Task |  | Pre-training | | Post-training | | Difference | | Pre-training | | Post-training | | Difference | | Pre-training | | Post-training | | Difference | |
|  |  | M | SD | M | SD | M | SD | M | SD | M | SD | M | SD | M | SD | M | SD | M | SD |
|  | Ori-CDT | 1198 | 359 | 709 | 146 | -489 | 342 | 1089 | 363 | 701 | 144 | -388 | 343 | 1307 | 364 | 717 | 150 | -590 | 351 |
| Ori-CDT | Col-CDT | 1117 | 367 | 963 | 193 | -155 | 346 | 1006 | 352 | 958 | 199 | -48 | 339 | 1229 | 388 | 967 | 191 | -261 | 362 |
|  | Dual-CDT | 1132 | 391 | 780 | 167 | -353 | 349 | 1038 | 375 | 779 | 163 | -258 | 338 | 1227 | 413 | 780 | 173 | -447 | 369 |
|  | Digit-Span | 1223 | 373 | 1030 | 293 | -193 | 219 | 1102 | 371 | 1024 | 295 | -77 | 212 | 1344 | 384 | 1035 | 294 | -308 | 239 |
|  | Ori-CDT | 1839 | 472 | 1345 | 375 | -494 | 496 | 1759 | 442 | 1283 | 350 | -476 | 472 | 1920 | 507 | 1407 | 406 | -513 | 526 |
|  | Col-CDT | 1781 | 456 | 1339 | 238 | -442 | 435 | 1702 | 437 | 1251 | 217 | -451 | 429 | 1861 | 483 | 1427 | 270 | -434 | 453 |
| Col-CDT | Dual-CDT | 1633 | 455 | 1246 | 288 | -386 | 465 | 1564 | 431 | 1183 | 281 | -381 | 451 | 1702 | 489 | 1310 | 301 | -392 | 490 |
|  | Digit-Span | 1834 | 529 | 1540 | 511 | -293 | 433 | 1752 | 510 | 1450 | 476 | -303 | 393 | 1915 | 553 | 1631 | 552 | -284 | 481 |
|  | Ori-CDT | 930 | 268 | 638 | 182 | -293 | 195 | 869 | 269 | 605 | 177 | -265 | 204 | 991 | 271 | 670 | 190 | -321 | 191 |
| Dual-Ori- CDT | Col-CDT | 1018 | 347 | 842 | 234 | -177 | 245 | 961 | 345 | 791 | 244 | -170 | 254 | 1075 | 358 | 893 | 232 | -183 | 245 |
|  | Dual-CDT | 918 | 295 | 610 | 193 | -309 | 252 | 862 | 297 | 571 | 182 | -292 | 250 | 975 | 300 | 649 | 210 | -326 | 266 |
|  | Digit-Span | 929 | 353 | 760 | 259 | -168 | 190 | 856 | 344 | 709 | 249 | -147 | 193 | 1001 | 368 | 812 | 274 | -189 | 202 |
|  | Ori-CDT | 1442 | 425 | 1244 | 338 | -198 | 354 | 1419 | 436 | 1230 | 333 | -189 | 371 | 1465 | 420 | 1258 | 348 | -207 | 348 |
| Dual-Col- CDT | Col-CDT | 1580 | 448 | 1311 | 294 | -269 | 344 | 1567 | 437 | 1272 | 277 | -295 | 345 | 1593 | 466 | 1350 | 318 | -243 | 355 |
|  | Dual-CDT | 1504 | 466 | 1094 | 309 | -410 | 430 | 1480 | 467 | 1067 | 299 | -413 | 418 | 1528 | 469 | 1121 | 328 | -407 | 454 |
|  | Digit-Span | 1494 | 507 | 1355 | 517 | -139 | 262 | 1451 | 482 | 1325 | 494 | -126 | 265 | 1537 | 540 | 1384 | 546 | -153 | 278 |

| Table S4. Summary statistics for change detection accuracy performance across cue-type pre and post, split by set-size. | | | | | | | | | | | | | | | | | | | |
| --- | --- | --- | --- | --- | --- | --- | --- | --- | --- | --- | --- | --- | --- | --- | --- | --- | --- | --- | --- |
| Accuracy (%) | | | | | | | | | | | | | | | | | | | |
| Task | Training  group |  |  | Set-size 2 | |  |  |  |  | Set-Size 4 | |  |  |  |  | Set-size 8 | |  |  |
|  |  | Pre-training | | Post-training | | Difference | | Pre-training | | Post-training | | Difference | | Pre-training | | Post-training | | Difference | |
|  |  | M | SD | M | SD | M | SD | M | SD | M | SD | M | SD | M | SD | M | SD | M | SD |
|  | Ori-CDT | 77.78 | 10.74 | 84.64 | 6.78 | 6.86 | 10.97 | 66.67 | 10.39 | 65.36 | 8.04 | -1.31 | 9.54 | 57.30 | 8.11 | 59.07 | 8.03 | 1.77 | 9.05 |
| Ori-CDT | Col-CDT | 75.63 | 11.77 | 79.86 | 10.51 | 4.23 | 10.48 | 65.18 | 9.72 | 62.57 | 8.71 | -2.61 | 9.57 | 55.89 | 6.85 | 56.99 | 7.85 | 1.1 | 7.43 |
|  | Dual-CDT | 76.10 | 10.5 | 82.74 | 9.53 | 6.65 | 10.04 | 65.89 | 8.80 | 65.28 | 7.74 | -0.61 | 10.90 | 56.26 | 6.73 | 58.8 | 8.01 | 2.54 | 8.04 |
|  | Digit-Span | 80.38 | 10.66 | 82.23 | 8.49 | 1.86 | 9.07 | 67.65 | 10.12 | 63.83 | 9.25 | -3.83 | 9.16 | 57.80 | 7.29 | 57.01 | 6.47 | -0.80 | 7.20 |
|  | Ori-CDT | 73.81 | 12.45 | 75.48 | 10.38 | 1.67 | 11.48 | 64.48 | 9.89 | 68.77 | 9.28 | 4.29 | 11.13 | 55.71 | 8.35 | 57.82 | 8.28 | 2.10 | 10.37 |
|  | Col-CDT | 71.83 | 13.80 | 84.92 | 9.74 | 13.09 | 13.75 | 65.89 | 10.99 | 76.18 | 10.61 | 10.29 | 13.69 | 56.83 | 8.71 | 65.61 | 12.19 | 8.78 | 14.26 |
| Col-CDT | Dual-CDT | 71.87 | 10.89 | 81.54 | 10.00 | 9.67 | 11.92 | 67.28 | 9.34 | 73.33 | 9.91 | 6.06 | 9.98 | 57.36 | 7.39 | 60.75 | 10.13 | 3.40 | 10.43 |
|  | Digit-Span | 74.09 | 11.27 | 79.60 | 9.73 | 5.51 | 12.40 | 67.54 | 9.13 | 69.20 | 9.73 | 1.67 | 10.71 | 56.59 | 6.51 | 60.19 | 7.92 | 3.60 | 8.93 |
|  | Ori-CDT | 70.44 | 9.95 | 77.91 | 7.38 | 7.47 | 7.96 | 61.67 | 9.24 | 69.54 | 7.56 | 7.87 | 8.31 | 55.03 | 6.43 | 60.55 | 7.85 | 5.52 | 6.96 |
| Dual-Ori- CDT | Col-CDT | 69.55 | 10.97 | 76.76 | 9.62 | 7.22 | 9.90 | 62.74 | 9.69 | 67.95 | 9.85 | 5.22 | 6.32 | 55.39 | 7.74 | 58.88 | 6.80 | 3.49 | 7.72 |
|  | Dual-CDT | 70.49 | 12.12 | 78.71 | 7.83 | 8.21 | 11.53 | 63.82 | 8.92 | 70.16 | 8.64 | 6.33 | 8.34 | 55.89 | 7.01 | 60.06 | 7.22 | 4.17 | 9.85 |
|  | Digit-Span | 74.46 | 9.25 | 77.81 | 9.92 | 3.35 | 8.56 | 62.94 | 10.19 | 65.15 | 9.71 | 2.21 | 9.00 | 58.30 | 7.66 | 57.45 | 7.63 | -0.85 | 8.37 |
|  | Ori-CDT | 67.39 | 9.62 | 71.03 | 10.35 | 3.64 | 8.96 | 60.91 | 9.88 | 63.1 | 9.41 | 2.18 | 10.75 | 55.92 | 6.65 | 57.77 | 7.74 | 1.85 | 8.22 |
| Dual-Col- CDT | Col-CDT | 68.50 | 11.77 | 78.59 | 10.85 | 10.09 | 10.34 | 60.60 | 10.21 | 71.34 | 12.12 | 10.74 | 9.89 | 55.39 | 7.16 | 60.94 | 8.81 | 5.56 | 9.02 |
|  | Dual-CDT | 69.75 | 10.51 | 80.00 | 9.23 | 10.25 | 10.04 | 69.75 | 60.40 | 9.12 | 69.8 | 10.13 | 9.40 | 55.69 | 7.94 | 60.23 | 7.96 | 4.54 | 9.01 |
|  | Digit-Span | 69.51 | 11.19 | 74.12 | 11.11 | 4.61 | 11.19 | 61.90 | 10.16 | 64.43 | 11.10 | 2.53 | 10.83 | 55.21 | 5.89 | 57.99 | 7.36 | 2.78 | 7.02 |

| Table S5. Summary statistics for change detection reaction time performance across cue-type pre and post, split by set-size. | | | | | | | | | | | | | | | | | | | |
| --- | --- | --- | --- | --- | --- | --- | --- | --- | --- | --- | --- | --- | --- | --- | --- | --- | --- | --- | --- |
| Reaction time (ms) | | | | | | | | | | | | | | | | | | | |
|  | Training group |  |  | Set-size 2 | |  |  |  |  | Set-Size 4 | |  |  |  |  | Set-size 8 | |  |  |
| Task |  | Pre-training | | Post-training | | Difference | | Pre-training | | Post-training | | Difference | | Pre-training | | Post-training | | Difference | |
|  |  | M | SD | M | SD | M | SD | M | SD | M | SD | M | SD | M | SD | M | SD | M | SD |
|  | Ori-CDT | 1250 | 346 | 668 | 135 | -581 | 346 | 1237 | 390 | 730 | 155 | -508 | 376 | 1107 | 416 | 729 | 167 | -379 | 372 |
| Ori-CDT | Col-CDT | 1147 | 416 | 941 | 192 | -206 | 413 | 1141 | 375 | 990 | 203 | -151 | 355 | 1064 | 366 | 957 | 237 | -107 | 317 |
|  | Dual-CDT | 1151 | 407 | 748 | 162 | -402 | 366 | 1143 | 409 | 788 | 177 | -354 | 368 | 1104 | 384 | 803 | 183 | -302 | 346 |
|  | Digit-Span | 1244 | 365 | 1039 | 289 | -204 | 236 | 1236 | 369 | 1057 | 299 | -179 | 236 | 1189 | 449 | 993 | 327 | -196 | 260 |
|  | Ori-CDT | 2068 | 422 | 1472 | 356 | -597 | 483 | 1836 | 507 | 1352 | 378 | -483 | 534 | 1615 | 558 | 1212 | 425 | -404 | 533 |
|  | Col-CDT | 1942 | 476 | 1349 | 249 | -594 | 444 | 1791 | 476 | 1346 | 248 | -444 | 487 | 1613 | 524 | 1321 | 308 | -292 | 444 |
| Col-CDT | Dual-CDT | 1788 | 458 | 1279 | 263 | -509 | 456 | 1631 | 470 | 1249 | 286 | -382 | 490 | 1480 | 505 | 1212 | 337 | -268 | 511 |
|  | Digit-Span | 2012 | 497 | 1657 | 483 | -354 | 432 | 1836 | 523 | 1553 | 519 | -282 | 450 | 1654 | 637 | 1411 | 580 | -244 | 473 |
|  | Ori-CDT | 1007 | 273 | 634 | 183 | -373 | 215 | 931 | 287 | 637 | 180 | -294 | 209 | 853 | 286 | 642 | 199 | -211 | 212 |
| Dual-Ori- CDT | Col-CDT | 1062 | 352 | 854 | 273 | -208 | 263 | 1027 | 344 | 843 | 224 | -185 | 253 | 966 | 391 | 828 | 243 | -138 | 272 |
|  | Dual-CDT | 958 | 323 | 605 | 179 | -353 | 278 | 915 | 295 | 610 | 203 | -305 | 260 | 882 | 295 | 614 | 209 | -268 | 252 |
|  | Digit-Span | 996 | 371 | 801 | 275 | -195 | 210 | 922 | 346 | 759 | 261 | -163 | 197 | 868 | 386 | 721 | 272 | -147 | 212 |
|  | Ori-CDT | 1667 | 438 | 1363 | 326 | -304 | 387 | 1416 | 441 | 1231 | 329 | -186 | 353 | 1243 | 457 | 1138 | 380 | -104 | 393 |
| Dual-Col- CDT | Col-CDT | 1754 | 453 | 1384 | 308 | -370 | 331 | 1569 | 463 | 1310 | 309 | -259 | 374 | 1418 | 535 | 1239 | 371 | -178 | 398 |
|  | Dual-CDT | 1653 | 468 | 1148 | 284 | -505 | 420 | 1498 | 485 | 1089 | 321 | -409 | 464 | 1361 | 489 | 1044 | 350 | -317 | 469 |
|  | Digit-Span | 1675 | 490 | 1517 | 497 | -158 | 333 | 1466 | 530 | 1340 | 528 | -126 | 282 | 1342 | 577 | 1208 | 587 | -134 | 252 |

| Table S6. Summary statistics for the orientation-CDT accuracy performance split by group, set-size, and cue-type. | | | | | | | | | | | | | | | | | | | |
| --- | --- | --- | --- | --- | --- | --- | --- | --- | --- | --- | --- | --- | --- | --- | --- | --- | --- | --- | --- |
| Accuracy (%) | | | | | | | | | | | | | | | | | | | |
| Training  group | Set-  size |  |  | Across cue-type | |  |  |  |  | Cue |  |  |  |  |  | No-cue | |  |  |
|  |  | Pre-training | | Post-training | | Difference | | Pre-training | | Post-training | | Difference | | Pre-training | | Post-training | | Difference | |
|  |  | M | SD | M | SD | M | SD | M | SD | M | SD | M | SD | M | SD | M | SD | M | SD |
|  | Total | 67.25 | 8.28 | 69.69 | 6.40 | 2.44 | 7.25 | 69.97 | 10.31 | 70.98 | 7.21 | 1.00 | 8.74 | 64.52 | 7.63 | 68.4 | 6.78 | 3.87 | 7.94 |
| Ori-CDT | 2 | 77.78 | 10.74 | 84.64 | 6.78 | 6.86 | 10.97 | 79.44 | 12.86 | 87.22 | 7.86 | 7.78 | 12.96 | 76.11 | 11.47 | 82.06 | 7.51 | 5.95 | 12.66 |
|  | 4 | 66.67 | 10.39 | 65.36 | 8.04 | -1.31 | 9.54 | 68.02 | 14.24 | 62.50 | 10.39 | -5.52 | 14.22 | 65.32 | 10.36 | 68.21 | 8.75 | 2.90 | 11.76 |
|  | 8 | 57.30 | 8.11 | 59.07 | 8.03 | 1.77 | 9.05 | 62.46 | 11.29 | 63.21 | 10.71 | 0.75 | 12.14 | 52.14 | 9.82 | 54.92 | 9.17 | 2.78 | 10.87 |
|  | Total | 65.57 | 8.32 | 66.48 | 7.94 | 0.91 | 7.01 | 68.44 | 10.5 | 67.48 | 8.79 | -0.97 | 8.61 | 62.70 | 7.66 | 65.47 | 8.02 | 2.78 | 8.08 |
|  | 2 | 75.63 | 11.77 | 79.86 | 10.51 | 4.23 | 10.48 | 77.32 | 14.89 | 81.91 | 10.36 | 4.59 | 12.39 | 73.94 | 11.77 | 77.8 | 12.26 | 3.86 | 12.37 |
| Col-CDT | 4 | 65.18 | 9.72 | 62.57 | 8.71 | -2.61 | 9.57 | 67.56 | 11.28 | 60.84 | 10.41 | -6.72 | 12.94 | 62.81 | 10.93 | 64.31 | 9.43 | 1.50 | 10.85 |
|  | 8 | 55.89 | 6.85 | 56.99 | 7.85 | 1.1 | 7.43 | 60.45 | 11.8 | 59.68 | 10.61 | -0.77 | 12.18 | 51.34 | 6.88 | 54.31 | 9.44 | 2.97 | 10.94 |
|  | Total | 66.08 | 7.27 | 68.94 | 6.9 | 2.86 | 7.18 | 70.22 | 9.47 | 70.26 | 8.43 | 0.04 | 9.60 | 61.95 | 6.17 | 67.63 | 6.87 | 5.68 | 6.97 |
|  | 2 | 76.10 | 10.5 | 82.74 | 9.53 | 6.65 | 10.04 | 80.49 | 11.27 | 84.59 | 10.66 | 4.11 | 12.94 | 71.71 | 11.01 | 80.89 | 10.17 | 9.19 | 10.77 |
| Dual-CDT | 4 | 65.89 | 8.80 | 65.28 | 7.74 | -0.61 | 10.90 | 69.02 | 11.91 | 63.01 | 10.46 | -6.02 | 14.3 | 62.76 | 8.5 | 67.56 | 7.99 | 4.80 | 10.25 |
|  | 8 | 56.26 | 6.73 | 58.8 | 8.01 | 2.54 | 8.04 | 61.14 | 10.4 | 63.17 | 11.1 | 2.03 | 11.13 | 51.38 | 7.67 | 54.43 | 9.41 | 3.05 | 11.5 |
|  | Total | 68.61 | 8.01 | 67.69 | 6.74 | -0.92 | 6.21 | 73.01 | 10.04 | 69.65 | 8.23 | -3.36 | 7.71 | 64.22 | 7.33 | 65.73 | 6.58 | 1.51 | 6.67 |
| Digit-Span | 2 | 80.38 | 10.66 | 82.23 | 8.49 | 1.86 | 9.07 | 83.03 | 13.9 | 85.3 | 8.14 | 2.27 | 11.7 | 77.73 | 9.72 | 79.17 | 10.86 | 1.44 | 11.24 |
|  | 4 | 67.65 | 10.12 | 63.83 | 9.25 | -3.83 | 9.16 | 72.88 | 10.87 | 61.97 | 12.79 | -10.91 | 11.19 | 62.42 | 11.47 | 65.68 | 8.55 | 3.26 | 11.38 |
|  | 8 | 57.80 | 7.29 | 57.01 | 6.47 | -0.80 | 7.20 | 63.11 | 10.96 | 61.67 | 9.30 | -1.44 | 10.77 | 52.5 | 8.78 | 52.35 | 8.52 | -0.15 | 10.37 |

| Table S7. Summary statistics for the colour-CDT accuracy performance split by group, set-size, and cue-type. | | | | | | | | | | | | | | | | | | | |
| --- | --- | --- | --- | --- | --- | --- | --- | --- | --- | --- | --- | --- | --- | --- | --- | --- | --- | --- | --- |
| Accuracy (%) | | | | | | | | | | | | | | | | | | | |
| Training  group | Set-  size |  |  | Across cue-type | |  |  |  |  | Cue |  |  |  |  |  | No-cue | |  |  |
|  |  | Pre-training | | Post-training | | Difference | | Pre-training | | Post-training | | Difference | | Pre-training | | Post-training | | Difference | |
|  |  | M | SD | M | SD | M | SD | M | SD | M | SD | M | SD | M | SD | M | SD | M | SD |
|  | Total | 64.67 | 8.15 | 67.35 | 7.37 | 2.69 | 8.17 | 66.38 | 9.22 | 71.06 | 7.50 | 4.68 | 10.25 | 62.96 | 8.32 | 63.65 | 8.71 | 0.69 | 7.80 |
|  | 2 | 73.81 | 12.45 | 75.48 | 10.38 | 1.67 | 11.48 | 73.97 | 14.75 | 79.21 | 10.33 | 5.24 | 13.34 | 73.65 | 12.92 | 71.75 | 12.5 | -1.90 | 12.96 |
| Ori-CDT | 4 | 64.48 | 9.89 | 68.77 | 9.28 | 4.29 | 11.13 | 67.46 | 11.04 | 72.46 | 10.12 | 5.00 | 14.32 | 61.51 | 12.03 | 65.08 | 12.54 | 3.57 | 14.25 |
|  | 8 | 55.71 | 8.35 | 57.82 | 8.28 | 2.10 | 10.37 | 57.70 | 9.41 | 61.51 | 9.46 | 3.81 | 13.1 | 53.73 | 11.90 | 54.13 | 11.54 | 0.40 | 14.56 |
|  | Total | 64.85 | 8.83 | 75.57 | 9.68 | 10.72 | 10.93 | 67.07 | 9.80 | 78.70 | 9.59 | 11.63 | 11.11 | 62.63 | 8.99 | 72.44 | 10.34 | 9.81 | 11.63 |
|  | 2 | 71.83 | 13.80 | 84.92 | 9.74 | 13.09 | 13.75 | 72.28 | 13.73 | 86.67 | 9.07 | 14.39 | 13.45 | 71.38 | 15.37 | 83.17 | 12.20 | 11.79 | 16.48 |
| Col-CDT | 4 | 65.89 | 10.99 | 76.18 | 10.61 | 10.29 | 13.69 | 68.54 | 13.15 | 82.19 | 10.16 | 13.66 | 14.10 | 63.25 | 11.46 | 70.16 | 12.58 | 6.91 | 16.72 |
|  | 8 | 56.83 | 8.71 | 65.61 | 12.19 | 8.78 | 14.26 | 60.41 | 11.79 | 67.24 | 14.16 | 6.83 | 17.59 | 53.25 | 9.44 | 63.98 | 12.00 | 10.73 | 13.65 |
|  | Total | 65.5 | 7.06 | 71.88 | 8.68 | 6.38 | 7.72 | 69.00 | 8.69 | 75.11 | 9.46 | 6.11 | 9.07 | 62.00 | 7.72 | 68.65 | 9.24 | 6.64 | 9.78 |
|  | 2 | 71.87 | 10.89 | 81.54 | 10.00 | 9.67 | 11.92 | 73.09 | 12.68 | 82.44 | 11.16 | 9.35 | 13.71 | 70.65 | 12.07 | 80.65 | 11.11 | 10.00 | 13.10 |
| Dual-CDT | 4 | 67.28 | 9.34 | 73.33 | 9.91 | 6.06 | 9.98 | 73.09 | 10.73 | 78.25 | 11.09 | 5.16 | 11.78 | 61.46 | 11.62 | 68.41 | 12.11 | 6.95 | 14.50 |
|  | 8 | 57.36 | 7.39 | 60.75 | 10.13 | 3.40 | 10.43 | 60.81 | 10.16 | 64.63 | 12.91 | 3.82 | 13.71 | 53.9 | 8.88 | 56.87 | 11.33 | 2.97 | 13.75 |
|  | Total | 66.07 | 7.43 | 69.67 | 7.14 | 3.59 | 7.31 | 67.85 | 8.38 | 72.71 | 8.51 | 4.86 | 9.22 | 64.29 | 7.54 | 66.62 | 7.03 | 2.32 | 7.37 |
|  | 2 | 74.09 | 11.27 | 79.60 | 9.73 | 5.51 | 12.40 | 73.79 | 13.09 | 80.15 | 10.39 | 6.36 | 13.62 | 74.39 | 11.56 | 79.05 | 10.99 | 4.66 | 14.09 |
| Digit-Span | 4 | 67.54 | 9.13 | 69.20 | 9.73 | 1.67 | 10.71 | 70.68 | 11.06 | 74.51 | 13.07 | 3.83 | 14.45 | 64.39 | 9.80 | 63.90 | 10.01 | -0.49 | 11.98 |
|  | 8 | 56.59 | 6.51 | 60.19 | 7.92 | 3.60 | 8.93 | 59.09 | 8.84 | 63.49 | 10.92 | 4.39 | 13.29 | 54.09 | 8.80 | 56.89 | 11.17 | 2.80 | 13.37 |

| Table S8. Summary statistics for the Dual-Orientation-CDT accuracy performance split by group, set-size, and cue-type. | | | | | | | | | | | | | | | | | | | |
| --- | --- | --- | --- | --- | --- | --- | --- | --- | --- | --- | --- | --- | --- | --- | --- | --- | --- | --- | --- |
|  |  |  |  |  |  |  |  |  |  | Accuracy (%) | |  |  |  |  |  |  |  |  |
| Training group | Set- size |  |  | Across cue-type | |  |  |  |  | Cue |  |  |  |  |  | No-cue | |  |  |
|  |  | Pre-training | | Post-training | | Difference | | Pre-training | | Post-training | | Difference | | Pre-training | | Post-training | | Difference | |
|  |  | M | SD | M | SD | M | SD | M | SD | M | SD | M | SD | M | SD | M | SD | M | SD |
|  | Total | 62.38 | 7.30 | 69.33 | 6.56 | 6.96 | 5.29 | 65.17 | 9.07 | 72.46 | 7.81 | 7.30 | 7.03 | 59.59 | 7.31 | 66.20 | 6.63 | 6.61 | 6.98 |
|  | 2 | 70.44 | 9.95 | 77.91 | 7.38 | 7.47 | 7.96 | 73.74 | 12.04 | 79.56 | 9.16 | 5.82 | 9.40 | 67.13 | 10.52 | 76.26 | 8.35 | 9.13 | 10.54 |
| Ori-CDT | 4 | 61.67 | 9.24 | 69.54 | 7.56 | 7.87 | 8.31 | 66.14 | 12.83 | 75.26 | 9.76 | 9.13 | 13.25 | 57.21 | 10.23 | 63.82 | 8.06 | 6.61 | 9.62 |
|  | 8 | 55.03 | 6.43 | 60.55 | 7.85 | 5.52 | 6.96 | 55.62 | 8.71 | 62.57 | 9.80 | 6.94 | 9.45 | 54.43 | 8.52 | 58.53 | 8.42 | 4.10 | 11.01 |
|  | Total | 62.56 | 7.94 | 67.86 | 7.8 | 5.31 | 5.82 | 65.33 | 9.81 | 71.09 | 8.97 | 5.76 | 7.85 | 59.78 | 7.52 | 64.63 | 7.88 | 4.85 | 6.07 |
|  | 2 | 69.55 | 10.97 | 76.76 | 9.62 | 7.22 | 9.90 | 72.15 | 12.83 | 79.13 | 11.08 | 6.98 | 11.08 | 66.94 | 11.58 | 74.39 | 10.51 | 7.45 | 13.06 |
| Col-CDT | 4 | 62.74 | 9.69 | 67.95 | 9.85 | 5.22 | 6.32 | 66.73 | 13.37 | 72.97 | 11.88 | 6.23 | 11.19 | 58.74 | 8.55 | 62.94 | 10.44 | 4.20 | 8.01 |
|  | 8 | 55.39 | 7.74 | 58.88 | 6.80 | 3.49 | 7.72 | 57.11 | 9.82 | 61.18 | 9.03 | 4.06 | 12.39 | 53.66 | 10.46 | 56.57 | 8.53 | 2.91 | 11.02 |
|  | Total | 63.40 | 8.13 | 69.64 | 6.54 | 6.24 | 7.85 | 67.12 | 9.71 | 73.14 | 8.05 | 6.02 | 9.48 | 59.69 | 7.38 | 66.15 | 6.62 | 6.46 | 8.23 |
|  | 2 | 70.49 | 12.12 | 78.71 | 7.83 | 8.21 | 11.53 | 74.05 | 12.84 | 81.3 | 8.52 | 7.25 | 11.75 | 66.94 | 14.08 | 76.12 | 10.00 | 9.18 | 14.58 |
| Dual-CDT | 4 | 63.82 | 8.92 | 70.16 | 8.64 | 6.33 | 8.34 | 68.63 | 12.75 | 77.27 | 10.69 | 8.64 | 11.91 | 59.01 | 8.62 | 63.04 | 8.75 | 4.03 | 9.18 |
|  | 8 | 55.89 | 7.01 | 60.06 | 7.22 | 4.17 | 9.85 | 58.67 | 10.08 | 60.84 | 10.33 | 2.17 | 12.86 | 53.12 | 8.88 | 59.28 | 8.66 | 6.17 | 13.26 |
|  | Total | 65.24 | 7.80 | 66.80 | 7.88 | 1.57 | 6.72 | 69.19 | 10.26 | 71.19 | 9.96 | 2.00 | 8.73 | 61.28 | 6.50 | 62.42 | 7.23 | 1.14 | 7.62 |
|  | 2 | 74.46 | 9.25 | 77.81 | 9.92 | 3.35 | 8.56 | 77.97 | 10.66 | 81.76 | 10.97 | 3.79 | 9.64 | 70.96 | 9.83 | 73.86 | 11.23 | 2.90 | 11.22 |
| Digit-Span | 4 | 62.94 | 10.19 | 65.15 | 9.71 | 2.21 | 9.00 | 68.88 | 13.85 | 72.28 | 13.72 | 3.41 | 13.76 | 57.01 | 9.27 | 58.02 | 9.53 | 1.01 | 10.50 |
|  | 8 | 58.30 | 7.66 | 57.45 | 7.63 | -0.85 | 8.37 | 60.73 | 10.49 | 59.53 | 10.52 | -1.20 | 10.10 | 55.87 | 8.56 | 55.37 | 8.33 | -0.50 | 11.31 |

| Table S9. Summary statistics for the Dual-Colour-CDT accuracy performance split by group, set-size, and cue-type. | | | | | | | | | | | | | | | | | | | |
| --- | --- | --- | --- | --- | --- | --- | --- | --- | --- | --- | --- | --- | --- | --- | --- | --- | --- | --- | --- |
|  |  |  |  |  |  |  |  |  |  | Accuracy (%) | |  |  |  |  |  |  |  |  |
| Training group | Set- size |  |  | Across cue-type | |  |  |  |  | Cue |  |  |  |  |  | No-cue | |  |  |
|  |  | Pre-training | | Post-training | | Difference | | Pre-training | | Post-training | | Difference | | Pre-training | | Post-training | | Difference | |
|  |  | M | SD | M | SD | M | SD | M | SD | M | SD | M | SD | M | SD | M | SD | M | SD |
|  | Total | 61.41 | 7.38 | 63.97 | 8.06 | 2.56 | 7.31 | 63.14 | 9.05 | 67.11 | 9.11 | 3.97 | 9.01 | 59.68 | 7.33 | 60.82 | 8.34 | 1.15 | 8.74 |
|  | 2 | 67.39 | 9.62 | 71.03 | 10.35 | 3.64 | 8.96 | 68.78 | 10.53 | 74.54 | 11.14 | 5.75 | 11.76 | 66.01 | 11.09 | 67.53 | 12.45 | 1.52 | 12.21 |
| Ori-CDT | 4 | 60.91 | 9.88 | 63.1 | 9.41 | 2.18 | 10.75 | 63.43 | 13.51 | 66.53 | 10.73 | 3.11 | 11.68 | 58.4 | 10.37 | 59.66 | 10.47 | 1.26 | 14.13 |
|  | 8 | 55.92 | 6.65 | 57.77 | 7.74 | 1.85 | 8.22 | 57.21 | 9.82 | 60.25 | 10.97 | 3.04 | 12.88 | 54.63 | 7.38 | 55.29 | 8.34 | 0.66 | 10.52 |
|  | Total | 61.50 | 8.42 | 70.29 | 9.58 | 8.80 | 7.63 | 63.87 | 9.73 | 74.21 | 9.52 | 10.34 | 8.69 | 59.12 | 8.09 | 66.37 | 10.39 | 7.25 | 8.67 |
|  | 2 | 68.50 | 11.77 | 78.59 | 10.85 | 10.09 | 10.34 | 70.33 | 13.14 | 81.17 | 9.85 | 10.84 | 10.61 | 66.67 | 12.16 | 76.02 | 13.73 | 9.35 | 13.25 |
| Col-CDT | 4 | 60.60 | 10.21 | 71.34 | 12.12 | 10.74 | 9.89 | 64.36 | 12.61 | 76.29 | 11.98 | 11.92 | 12.18 | 56.84 | 10.49 | 66.40 | 13.79 | 9.55 | 12.31 |
|  | 8 | 55.39 | 7.16 | 60.94 | 8.81 | 5.56 | 9.02 | 56.91 | 9.48 | 65.18 | 11.79 | 8.27 | 12.31 | 53.86 | 9.31 | 56.71 | 8.76 | 2.85 | 11.53 |
|  | Total | 61.95 | 7.77 | 70.01 | 7.83 | 8.06 | 7.25 | 64.90 | 9.02 | 74.23 | 9.17 | 9.33 | 9.32 | 58.99 | 7.57 | 65.79 | 7.71 | 6.80 | 6.97 |
|  | 2 | 69.75 | 10.51 | 80.00 | 9.23 | 10.25 | 10.04 | 71.48 | 11.32 | 82.86 | 9.46 | 11.38 | 12.59 | 68.02 | 12.41 | 77.13 | 11.83 | 9.11 | 11.41 |
| Dual-CDT | 4 | 60.40 | 9.12 | 69.8 | 10.13 | 9.40 | 9.10 | 64.50 | 10.73 | 74.29 | 12.6 | 9.79 | 12.85 | 56.3 | 10.63 | 65.31 | 11.84 | 9.01 | 12.76 |
|  | 8 | 55.69 | 7.94 | 60.23 | 7.96 | 4.54 | 9.01 | 58.74 | 10.84 | 65.55 | 10.6 | 6.81 | 12.49 | 52.64 | 8.58 | 54.91 | 8.38 | 2.27 | 11.04 |
|  | Total | 62.21 | 7.68 | 65.51 | 8.58 | 3.30 | 7.12 | 64.56 | 10.06 | 68.37 | 10.13 | 3.81 | 9.10 | 59.85 | 7.01 | 62.65 | 7.89 | 2.80 | 7.89 |
|  | 2 | 69.51 | 11.19 | 74.12 | 11.11 | 4.61 | 11.19 | 70.58 | 13.01 | 76.01 | 12.48 | 5.43 | 14.50 | 68.43 | 12.13 | 72.22 | 11.46 | 3.79 | 12.67 |
| Digit-Span | 4 | 61.90 | 10.16 | 64.43 | 11.10 | 2.53 | 10.83 | 65.28 | 12.55 | 67.68 | 14.00 | 2.40 | 12.84 | 58.52 | 10.10 | 61.17 | 11.17 | 2.65 | 12.42 |
|  | 8 | 55.21 | 5.89 | 57.99 | 7.36 | 2.78 | 7.02 | 57.83 | 9.72 | 61.43 | 8.99 | 3.60 | 9.63 | 52.59 | 7.20 | 54.55 | 9.07 | 1.96 | 11.20 |

| Table S10. Summary statistics for the orientation-CDT reaction time performance split by group, set-size, and cue-type. | | | | | | | | | | | | | | | | | | | |
| --- | --- | --- | --- | --- | --- | --- | --- | --- | --- | --- | --- | --- | --- | --- | --- | --- | --- | --- | --- |
| Accuracy (ms) | | | | | | | | | | | | | | | | | | | |
| Training  group | Set-  size |  |  | Across cue-type | |  |  |  |  | Cue |  |  |  |  |  | No-cue | |  |  |
|  |  | Pre-training | | Post-training | | Difference | | Pre-training | | Post-training | | Difference | | Pre-training | | Post-training | | Difference | |
|  |  | M | SD | M | SD | M | SD | M | SD | M | SD | M | SD | M | SD | M | SD | M | SD |
|  | Total | 1198 | 359 | 709 | 146 | -489 | 342 | 1089 | 363 | 701 | 144 | -388 | 343 | 1307 | 364 | 717 | 150 | -590 | 351 |
|  | 2 | 1250 | 346 | 668 | 135 | -581 | 346 | 1178 | 372 | 661 | 135 | -518 | 368 | 1322 | 342 | 676 | 140 | -645 | 344 |
| Ori-CDT | 4 | 1237 | 390 | 730 | 155 | -508 | 376 | 1114 | 393 | 721 | 153 | -393 | 377 | 1361 | 403 | 739 | 163 | -622 | 392 |
|  | 8 | 1107 | 416 | 729 | 167 | -379 | 372 | 975 | 413 | 720 | 167 | -255 | 365 | 1240 | 439 | 737 | 172 | -503 | 405 |
|  | Total | 1117 | 367 | 963 | 193 | -155 | 346 | 1006 | 352 | 958 | 199 | -48 | 339 | 1229 | 388 | 967 | 191 | -261 | 362 |
|  | 2 | 1147 | 416 | 941 | 192 | -206 | 413 | 1064 | 409 | 938 | 206 | -126 | 411 | 1231 | 431 | 944 | 184 | -287 | 425 |
| Col-CDT | 4 | 1141 | 375 | 990 | 203 | -151 | 355 | 1017 | 379 | 985 | 211 | -32 | 365 | 1265 | 386 | 995 | 209 | -271 | 369 |
|  | 8 | 1064 | 366 | 957 | 237 | -107 | 317 | 937 | 337 | 951 | 243 | 14 | 302 | 1190 | 412 | 963 | 239 | -227 | 357 |
|  | Total | 1132 | 391 | 780 | 167 | -353 | 349 | 1038 | 375 | 779 | 163 | -258 | 338 | 1227 | 413 | 780 | 173 | -447 | 369 |
|  | 2 | 1151 | 407 | 748 | 162 | -402 | 366 | 1093 | 424 | 752 | 157 | -340 | 376 | 1208 | 401 | 745 | 177 | -464 | 368 |
| Dual-CDT | 4 | 1143 | 409 | 788 | 177 | -354 | 368 | 1027 | 385 | 781 | 183 | -246 | 349 | 1258 | 445 | 796 | 176 | -463 | 402 |
|  | 8 | 1104 | 384 | 803 | 183 | -302 | 346 | 994 | 357 | 805 | 187 | -189 | 336 | 1215 | 426 | 801 | 186 | -414 | 375 |
|  | Total | 1223 | 373 | 1030 | 293 | -193 | 219 | 1102 | 371 | 1024 | 295 | -77 | 212 | 1344 | 384 | 1035 | 294 | -308 | 239 |
|  | 2 | 1244 | 365 | 1039 | 289 | -204 | 236 | 1136 | 377 | 1043 | 299 | -93 | 248 | 1351 | 363 | 1035 | 295 | -316 | 253 |
| Digit-Span | 4 | 1236 | 369 | 1057 | 299 | -179 | 236 | 1110 | 375 | 1052 | 311 | -58 | 221 | 1362 | 389 | 1063 | 299 | -300 | 296 |
|  | 8 | 1189 | 449 | 993 | 327 | -196 | 260 | 1059 | 430 | 978 | 322 | -81 | 278 | 1319 | 483 | 1008 | 338 | -311 | 289 |

| Table S11. Summary statistics for the colour-CDT reaction time performance split by group, set-size, and cue-type. | | | | | | | | | | | | | | | | | | | |
| --- | --- | --- | --- | --- | --- | --- | --- | --- | --- | --- | --- | --- | --- | --- | --- | --- | --- | --- | --- |
| Reaction time (ms) | | | | | | | | | | | | | | | | | | | |
| Training  group | Set-  size |  |  | Across cue-type | |  |  |  |  | Cue |  |  |  |  |  | No-cue | |  |  |
|  |  | Pre-training | | Post-training | | Difference | | Pre-training | | Post-training | | Difference | | Pre-training | | Post-training | | Difference | |
|  |  | M | SD | M | SD | M | SD | M | SD | M | SD | M | SD | M | SD | M | SD | M | SD |
|  | Total | 1839 | 472 | 1345 | 375 | -494 | 496 | 1759 | 442 | 1283 | 350 | -476 | 472 | 1920 | 507 | 1407 | 406 | -513 | 526 |
|  | 2 | 2068 | 422 | 1472 | 356 | -597 | 483 | 1977 | 400 | 1401 | 350 | -577 | 457 | 2159 | 457 | 1542 | 380 | -617 | 525 |
| Ori-CDT | 4 | 1836 | 507 | 1352 | 378 | -483 | 534 | 1816 | 495 | 1311 | 351 | -505 | 522 | 1856 | 539 | 1394 | 419 | -462 | 568 |
|  | 8 | 1615 | 558 | 1212 | 425 | -404 | 533 | 1484 | 533 | 1138 | 404 | -346 | 524 | 1747 | 596 | 1286 | 456 | -461 | 563 |
|  | Total | 1781 | 456 | 1339 | 238 | -442 | 435 | 1702 | 437 | 1251 | 217 | -451 | 429 | 1861 | 483 | 1427 | 270 | -434 | 453 |
|  | 2 | 1942 | 476 | 1349 | 249 | -594 | 444 | 1863 | 467 | 1275 | 259 | -588 | 456 | 2022 | 503 | 1423 | 266 | -599 | 459 |
| Col-CDT | 4 | 1791 | 476 | 1346 | 248 | -444 | 487 | 1740 | 470 | 1255 | 216 | -485 | 461 | 1841 | 498 | 1437 | 297 | -403 | 536 |
|  | 8 | 1613 | 524 | 1321 | 308 | -292 | 444 | 1505 | 515 | 1222 | 300 | -283 | 467 | 1721 | 559 | 1420 | 336 | -301 | 456 |
|  | Total | 1633 | 455 | 1246 | 288 | -386 | 465 | 1564 | 431 | 1183 | 281 | -381 | 451 | 1702 | 489 | 1310 | 301 | -392 | 490 |
|  | 2 | 1788 | 458 | 1279 | 263 | -509 | 456 | 1713 | 454 | 1210 | 263 | -502 | 457 | 1862 | 482 | 1347 | 275 | -516 | 482 |
| Dual-CDT | 4 | 1631 | 470 | 1249 | 286 | -382 | 490 | 1601 | 449 | 1199 | 281 | -402 | 474 | 1660 | 517 | 1299 | 309 | -361 | 534 |
|  | 8 | 1480 | 505 | 1212 | 337 | -268 | 511 | 1378 | 472 | 1139 | 328 | -238 | 500 | 1583 | 556 | 1284 | 354 | -299 | 544 |
|  | Total | 1834 | 529 | 1540 | 511 | -293 | 433 | 1752 | 510 | 1450 | 476 | -303 | 393 | 1915 | 553 | 1631 | 552 | -284 | 481 |
|  | 2 | 2012 | 497 | 1657 | 483 | -354 | 432 | 1908 | 472 | 1564 | 471 | -344 | 418 | 2116 | 541 | 1751 | 511 | -364 | 481 |
| Digit-Span | 4 | 1836 | 523 | 1553 | 519 | -282 | 450 | 1801 | 513 | 1477 | 462 | -324 | 413 | 1870 | 549 | 1630 | 589 | -240 | 513 |
|  | 8 | 1654 | 637 | 1411 | 580 | -244 | 473 | 1549 | 630 | 1309 | 566 | -239 | 440 | 1760 | 663 | 1512 | 605 | -248 | 529 |

| Table S12. Summary statistics for the Dual-Orientation-CDT reaction time performance split by group, set-size, and cue-type. | | | | | | | | | | | | | | | | | | | |
| --- | --- | --- | --- | --- | --- | --- | --- | --- | --- | --- | --- | --- | --- | --- | --- | --- | --- | --- | --- |
| Reaction time (ms) | | | | | | | | | | | | | | | | | | | |
| Training  group | Set-  size |  |  | Across cue-type | |  |  |  |  | Cue |  |  |  |  |  | No-cue | |  |  |
|  |  | Pre-training | | Post-training | | Difference | | Pre-training | | Post-training | | Difference | | Pre-training | | Post-training | | Difference | |
|  |  | M | SD | M | SD | M | SD | M | SD | M | SD | M | SD | M | SD | M | SD | M | SD |
|  | Total | 930 | 268 | 638 | 182 | -293 | 195 | 869 | 269 | 605 | 177 | -265 | 204 | 991 | 271 | 670 | 190 | -321 | 191 |
|  | 2 | 1007 | 273 | 634 | 183 | -373 | 215 | 941 | 274 | 603 | 183 | -338 | 233 | 1072 | 286 | 666 | 189 | -407 | 217 |
| Ori-CDT | 4 | 931 | 287 | 637 | 180 | -294 | 209 | 866 | 299 | 594 | 172 | -273 | 230 | 995 | 285 | 679 | 192 | -316 | 205 |
|  | 8 | 853 | 286 | 642 | 199 | -211 | 212 | 800 | 280 | 618 | 198 | -183 | 211 | 906 | 297 | 666 | 207 | -240 | 227 |
|  | Total | 1018 | 347 | 842 | 234 | -177 | 245 | 961 | 345 | 791 | 244 | -170 | 254 | 1075 | 358 | 893 | 232 | -183 | 245 |
|  | 2 | 1062 | 352 | 854 | 273 | -208 | 263 | 1006 | 357 | 809 | 293 | -197 | 284 | 1117 | 358 | 899 | 261 | -218 | 260 |
| Col-CDT | 4 | 1027 | 344 | 843 | 224 | -185 | 253 | 957 | 359 | 770 | 232 | -187 | 269 | 1098 | 349 | 915 | 237 | -182 | 255 |
|  | 8 | 966 | 391 | 828 | 243 | -138 | 272 | 920 | 369 | 793 | 248 | -128 | 274 | 1012 | 423 | 864 | 250 | -148 | 289 |
|  | Total | 918 | 295 | 610 | 193 | -309 | 252 | 862 | 297 | 571 | 182 | -292 | 250 | 975 | 300 | 649 | 210 | -326 | 266 |
|  | 2 | 958 | 323 | 605 | 179 | -353 | 278 | 898 | 336 | 566 | 168 | -333 | 283 | 1018 | 319 | 644 | 198 | -374 | 286 |
| Dual-CDT | 4 | 915 | 295 | 610 | 203 | -305 | 260 | 854 | 311 | 560 | 188 | -294 | 265 | 977 | 297 | 660 | 228 | -317 | 287 |
|  | 8 | 882 | 295 | 614 | 209 | -268 | 252 | 835 | 283 | 586 | 201 | -249 | 249 | 929 | 319 | 642 | 221 | -287 | 269 |
|  | Total | 929 | 353 | 760 | 259 | -168 | 190 | 856 | 344 | 709 | 249 | -147 | 193 | 1001 | 368 | 812 | 274 | -189 | 202 |
|  | 2 | 996 | 371 | 801 | 275 | -195 | 210 | 913 | 362 | 742 | 268 | -171 | 227 | 1078 | 390 | 860 | 293 | -218 | 222 |
| Digit-Span | 4 | 922 | 346 | 759 | 261 | -163 | 197 | 833 | 339 | 696 | 248 | -137 | 202 | 1012 | 363 | 822 | 288 | -190 | 230 |
|  | 8 | 868 | 386 | 721 | 272 | -147 | 212 | 822 | 375 | 689 | 262 | -134 | 208 | 913 | 406 | 754 | 293 | -160 | 241 |

| Table S13. Summary statistics for the Dual-Colour-CDT reaction time performance split by group, set-size, and cue-type. | | | | | | | | | | | | | | | | | | | |
| --- | --- | --- | --- | --- | --- | --- | --- | --- | --- | --- | --- | --- | --- | --- | --- | --- | --- | --- | --- |
| Reaction time (ms) | | | | | | | | | | | | | | | | | | | |
| Training  group | Set-  size |  |  | Across cue-type | |  |  |  |  | Cue |  |  |  |  |  | No-cue | |  |  |
|  |  | Pre-training | | Post-training | | Difference | | Pre-training | | Post-training | | Difference | | Pre-training | | Post-training | | Difference | |
|  |  | M | SD | M | SD | M | SD | M | SD | M | SD | M | SD | M | SD | M | SD | M | SD |
|  | Total | 1442 | 425 | 1244 | 338 | -198 | 354 | 1419 | 436 | 1230 | 333 | -189 | 371 | 1465 | 420 | 1258 | 348 | -207 | 348 |
| Ori-CDT | 2 | 1667 | 438 | 1363 | 326 | -304 | 387 | 1632 | 448 | 1328 | 312 | -304 | 388 | 1703 | 450 | 1398 | 350 | -304 | 423 |
|  | 4 | 1416 | 441 | 1231 | 329 | -186 | 353 | 1391 | 451 | 1223 | 327 | -167 | 370 | 1442 | 441 | 1238 | 349 | -204 | 359 |
|  | 8 | 1243 | 457 | 1138 | 380 | -104 | 393 | 1235 | 470 | 1140 | 391 | -95 | 436 | 1250 | 454 | 1137 | 379 | -113 | 364 |
|  | Total | 1580 | 448 | 1311 | 294 | -269 | 344 | 1567 | 437 | 1272 | 277 | -295 | 345 | 1593 | 466 | 1350 | 318 | -243 | 355 |
|  | 2 | 1754 | 453 | 1384 | 308 | -370 | 331 | 1729 | 461 | 1330 | 281 | -399 | 354 | 1779 | 463 | 1439 | 351 | -340 | 349 |
| Col-CDT | 4 | 1569 | 463 | 1310 | 309 | -259 | 374 | 1558 | 451 | 1270 | 296 | -288 | 403 | 1580 | 496 | 1350 | 344 | -230 | 377 |
|  | 8 | 1418 | 535 | 1239 | 371 | -178 | 398 | 1415 | 522 | 1218 | 366 | -198 | 391 | 1420 | 560 | 1261 | 396 | -159 | 442 |
|  | Total | 1504 | 466 | 1094 | 309 | -410 | 430 | 1480 | 467 | 1067 | 299 | -413 | 418 | 1528 | 469 | 1121 | 328 | -407 | 454 |
|  | 2 | 1653 | 468 | 1148 | 284 | -505 | 420 | 1607 | 492 | 1107 | 287 | -500 | 434 | 1698 | 454 | 1189 | 296 | -510 | 435 |
| Dual-CDT | 4 | 1498 | 485 | 1089 | 321 | -409 | 464 | 1463 | 497 | 1061 | 314 | -402 | 470 | 1534 | 489 | 1118 | 341 | -416 | 486 |
|  | 8 | 1361 | 489 | 1044 | 350 | -317 | 469 | 1372 | 474 | 1033 | 325 | -339 | 438 | 1351 | 519 | 1055 | 386 | -295 | 517 |
|  | Total | 1494 | 507 | 1355 | 517 | -139 | 262 | 1451 | 482 | 1325 | 494 | -126 | 265 | 1537 | 540 | 1384 | 546 | -153 | 278 |
| Digit-Span | 2 | 1675 | 490 | 1517 | 497 | -158 | 333 | 1616 | 474 | 1467 | 467 | -149 | 341 | 1735 | 520 | 1568 | 538 | -167 | 359 |
|  | 4 | 1466 | 530 | 1340 | 528 | -126 | 282 | 1442 | 501 | 1317 | 514 | -124 | 286 | 1490 | 573 | 1363 | 558 | -128 | 319 |
|  | 8 | 1342 | 577 | 1208 | 587 | -134 | 252 | 1296 | 553 | 1193 | 565 | -103 | 259 | 1389 | 613 | 1224 | 619 | -165 | 282 |

*ANCOVA results and pot-hoc follow ups*

| Table S14. ANCOVAs testing for main effects and interactions on accuracy | | | | | |
| --- | --- | --- | --- | --- | --- |
| Task | Main effects and Interactions |  | ANCOVA | |  |
|  |  | *df* | *F* | *p* | 𝜂2  𝑝 |
|  | Group | (3,983) | 5.703 | <0.001*** | 0.017 |
|  | Set-size | (2,983) | 234.960 | <0.001*** | 0.323 |
|  | Cue-type | (1,983) | 0.621 | 0.430 | 0.000 |
| Ori-CDT | Group*Set-size | (6,983) | 0.311 | 0.931 | 0.001 |
|  | Group*Cue-type | (3,983) | 0.211 | 0.889 | 0.000 |
|  | Set-size*Cue-type | (2,983) | 35.231 | <0.001*** | 0.066 |
|  | Group*Set-size*Cue-type | (6,983) | 0.621 | 0.713 | 0.003 |
|  | Group | (3,983) | 25.239 | <0.001*** | 0.071 |
|  | Set-size | (2,983) | 118.545 | <0.001*** | 0.194 |
|  | Cue-type | (1,983) | 57.572 | <0.001*** | 0.055 |
| Col-CDT | Group*Set-size | (6,983) | 0.995 | 0.427 | 0.006 |
|  | Group*Cue-type | (3,983) | 0.328 | 0.805 | 0.001 |
|  | Set-size*Cue-type | (2,983) | 4.170 | 0.015* | 0.008 |
|  | Group*Set-size*Cue-type | (6,983) | 1.396 | 0.212 | 0.008 |
|  | Group | (3,983) | 8.876 | <0.001*** | 0.026 |
|  | Set-size | (2,983) | 128.751 | <0.001*** | 0.207 |
|  | Cue-type | (1,983) | 60.377 | <0.001*** | 0.057 |
| Dual-Ori-CDT | Group*Set-size | (6,983) | 0.592 | 0.736 | 0.003 |
|  | Group*Cue-type | (3,983) | 0.484 | 0.693 | 0.001 |
|  | Set-size*Cue-type | (2,983) | 13.807 | <0.001*** | 0.027 |
|  | Group*Set-size*Cue-type | (6,983) | 0.968 | 0.445 | 0.005 |
|  | Group | (3,983) | 23.983 | <0.001*** | 0.068 |
|  | Set-size | (2,983) | 81.987 | <0.001*** | 0.143 |
|  | Cue-type | (1,983) | 60.745 | <0.001*** | 0.058 |
| Dual-Col-CDT | Group*Set-size | (6,983) | 1.795 | 0.096 | 0.010 |
|  | Group*Cue-type | (3,983) | 0.656 | 0.579 | 0.002 |
|  | Set-size*Cue-type | (2,983) | 0.662 | 0.516 | 0.001 |
|  | Group*Set-size*Cue-type | (6,983) | 0.398 | 0.880 | 0.002 |
| Digit-Span | Group | (3,162) | 31.661 | <0.001*** | 0.369 |
| *Note.* *p < .05. **p < .01. ***p < .001. | |  |  |  |  |

| Table S15. Cue-type comparisons of the adjusted whole task mean accuracy differences adjusted for  baseline performance. | | | | | | |
| --- | --- | --- | --- | --- | --- | --- |
| Task | Cue-type contrast | Post-training  accuracy difference (%) |  |  | t-test |  |
|  |  |  | *df* | *t* | *p* | 𝑑 |
| Ori-CDT | Cue-no-cue | 0.04 | 334 | 0.788 | 0.430 | 0.032 |
| Col-CDT | Cue-no-cue | 0.53 | 334 | 7.587 | <0.001*** | 0.378 |
| Dual-Ori | Cue-no-cue | 0.46 | 334 | 7.770 | <0.001*** | 0.366 |
| Dual-Col | Cue-no-cue | 0.51 | 334 | 7.793 | <0.001*** | 0.441 |
| *Note.* *p < .05. **p < .01. ***p < .001 (holm-corrected). | | | |  |  |  |

| Table S16. Pairwise set size comparisons of the adjusted whole task mean accuracy differences  adjusted for baseline performance. | | | | | | |
| --- | --- | --- | --- | --- | --- | --- |
| Task | Set-size contrast | Post-training  accuracy difference (%) |  |  | t-test |  |
|  |  |  | *df* | *t* | *p* | 𝑑 |
|  | Two-Four | 14.48 | 334 | 18.902 | <0.001*** | 1.423 |
| Ori-CDT | Two-Eight | 17.67 | 334 | 19.814 | <0.001*** | 0.307 |
|  | Four-Eight | 3.19 | 334 | 4.243 | <0.001*** | 1.703 |
|  | Two-Four | 6.84 | 334 | 7.806 | <0.001*** | 0.558 |
| Col-CDT | Two-Eight | 15.16 | 334 | 15.389 | <0.001*** | 1.262 |
|  | Four-Eight | 8.32 | 334 | 9.227 | <0.001*** | 0.659 |
|  | Two-Four | 6.44 | 334 | 8.979 | <0.001*** | 0.565 |
| Dual-Ori | Two-Eight | 12.92 | 334 | 16.043 | <0.001*** | 1.305 |
|  | Four-Eight | 6.48 | 334 | 8.979 | <0.001*** | 0.590 |
|  | Two-Four | 5.63 | 334 | 6.778 | <0.001*** | 0.485 |
| Dual-Col | Two-Eight | 11.39 | 334 | 12.798 | <0.001*** | 0.994 |
|  | Four-Eight | 5.76 | 334 | 7.095 | <0.001*** | 0.485 |
| Note. *p < .05. **p < .01. ***p < .001 (holm-corrected). | | | |  |  |  |

| Table S17. ANCOVAs testing for main effects and interactions on reaction time | | | | | |
| --- | --- | --- | --- | --- | --- |
| Task | Main effects and Interactions | ANCOVA | | | |
|  |  | *df* | *F* | *p* | 𝜂2  𝑝 |
|  | Group | (3,983) | 150.37 | <0.001*** | 0.314 |
|  | Set-size | (2,983) | 5.740 | <0.01** | 0.011 |
|  | Cue-type | (1,983) | 16.440 | <0.001*** | 0.016 |
| Ori-CDT | Group*Set-size | (6,983) | 1.886 | 0.080 | 0.001 |
|  | Group*Cue-type | (3,983) | 0.046 | 0.986 | 0.000 |
|  | Set-size*Cue-type | (2,983) | 0.110 | 0.895 | 0.000 |
|  | Group*Set-size*Cue-type | (6,983) | 0.108 | 0.995 | 0.000 |
|  | Group | (3,983) | 21.614 | <0.001*** | 0.061 |
|  | Set-size | (2,983) | 0.568 | 0.566 | 0.001 |
|  | Cue-type | (1,983) | 19.387 | <0.001*** | 0.019 |
| Col-CDT | Group*Set-size | (6,983) | 1.816 | 0.092 | 0.011 |
|  | Group*Cue-type | (3,983) | 0.416 | 0.740 | 0.001 |
|  | Set-size*Cue-type | (2,983) | 0.028 | 0.972 | 0.000 |
|  | Group*Set-size*Cue-type | (6,983) | 0.042 | 0.999 | 0.000 |
|  | Group | (3,983) | 69.599 | <0.001*** | 0.175 |
|  | Set-size | (2,983) | 2.703 | 0.067 | 0.005 |
|  | Cue-type | (1,983) | 7.045 | <0.01** | 0.007 |
| Dual-Ori-CDT | Group*Set-size | (6,983) | 1.294 | 0.256 | 0.007 |
|  | Group*Cue-type | (3,983) | 0.600 | 0.614 | 0.001 |
|  | Set-size*Cue-type | (2,983) | 0.737 | 0.478 | 0.001 |
|  | Group*Set-size*Cue-type | (6,983) | 0.062 | 0.999 | 0.000 |
|  | Group | (3,983) | 32.744 | <0.001*** | 0.090 |
|  | Set-size | (2,983) | 0.175 | 0.839 | 0.000 |
|  | Cue-type | (1,983) | 1.986 | 0.159 | 0.002 |
| Dual-Col-CDT | Group*Set-size | (6,983) | 1.539 | 0.161 | 0.009 |
|  | Group*Cue-type | (3,983) | 0.453 | 0.715 | 0.001 |
|  | Set-size*Cue-type | (2,983) | 0.285 | 0.751 | 0.000 |
|  | Group*Set-size*Cue-type | (6,983) | 0.051 | 0.999 | 0.000 |
| *Note.* *p < .05. **p < .01. ***p < .001. | |  |  |  |  |

| Table S18. Cue-type comparisons of the adjusted whole task mean reaction time differences  adjusted for baseline performance. | | | | | | | |
| --- | --- | --- | --- | --- | --- | --- | --- |
|  | Cue-type contrast | Post-training reaction time difference  (ms) | t-test | | | |  |
| Task |  |  |  |  |  |  |  |
|  |  |  | *df* | *t* | *p* | 𝑑 | |
| Ori-CDT | Cue-no-cue | -51.66 | 334 | 4.054 | <0.001*** | 0.199 | |
| Col-CDT | Cue-no-cue | -99.45 | 334 | 4.403 | <0.001*** | 0.240 | |
| Dual-Ori | Cue-no-cue | -29.18 | 334 | 2.654 | <0.01** | 0.116 | |
| Dual-Col | Cue-no-cue | -27.64 | 334 | 1.409 | 0.159 | 0.065 | |
| *Note.* *p < .05. **p < .01. ***p < .001 (holm-corrected). | | | |  |  |  | |

| Table S19. Pairwise set size comparisons of the adjusted whole task mean reaction time  differences adjusted for baseline performance. | | | | | | | |
| --- | --- | --- | --- | --- | --- | --- | --- |
|  | Set-size contrast | Post-training reaction time difference  (ms) |  |  | t-test | |  |
| Task |  |  |  |  |  |  |  |
|  |  |  | *df* | *t* | *p* | | 𝑑 |
|  | Two-Four | 44.44 | 334 | 2.954 | <0.01** | | 1.728 |
| Ori-CDT | Two-Eight | 43.96 | 334 | 2.912 | <0.01** | | 0.169 |
|  | Four-Eight | 0.48 | 334 | 0.031 | 0.974 | | 0.001 |
|  | Two-Four | 2.94 | 334 | 0.106 | 1.00 | | 0.886 |
| Col-CDT | Two-Eight | 27.44 | 334 | 0.964 | 1.00 | | 0.651 |
|  | Four-Eight | 24.50 | 334 | 0.886 | 1.00 | | 0.057 |
|  | Two-Four | 15.14 | 334 | 1.141 | 0.460 | | 0.059 |
| Dual-Ori | Two-Eight | 31.08 | 334 | 2.325 | 0.060 | | 0.121 |
|  | Four-Eight | 15.94 | 334 | 1.200 | 0.460 | | 0.059 |
|  | Two-Four | 6.15 | 334 | 0.253 | 1.00 | | 0.015 |
| Dual-Col | Two-Eight | 14.70 | 334 | 0.588 | 1.00 | | 0.035 |
|  | Four-Eight | 8.54 | 334 | 0.353 | 1.00 | | 0.020 |
| *Note.* *p < .05. **p < .01. ***p < .001 (holm-corrected). | | | |  |  | |  |
